# Hypoxia-induced genome-wide DNA demethylation by DNMT3A and EMT of cancer cells

**DOI:** 10.1186/s11658-025-00775-x

**Published:** 2025-08-05

**Authors:** Biswanath Chatterjee, Pritha Majumder, Chun-Chang Chen, Jing-Ping Wang, Po-Hsuan Su, Hung-Cheng Lai, Ching-Chen Liu, Hsin-Nan Lin, Chen-Hsin A. Yu, Hanna S. Yuan, Che-Kun James Shen

**Affiliations:** 1 The PhD Program in Medical Neuroscience, Taipei Medical University, 12F, Education & Research Building, Shuang-Ho Campus, No. 301, Yuantong Road, Zhonghe District, New Taipei City, 235 Taiwan; 2 Institute of Molecular Biology, Academia Sinica, No. 128, Section 2, Academia Rd, Nangang District, Taipei City, 115 Taiwan; 3 National Applied Research Laboratories, National Laboratory Animal Center, Building G, No. 111, Lane 130, Section 1, Academia Road, Nangang District, Taipei City, 115021 Taiwan; 4 Translational Epigenetics Center, Shuang Ho Hospital, Taipei Medical University, New Taipei City, 235 Taiwan

**Keywords:** Hypoxia, EMT of cancer cells, Epigenetic regulation, DNA demethylation, DNMT3A, Histone modifications

## Abstract

**Background:**

Despite the comprehensive advancement in the field of cancer therapeutics, there remains an urgent need to identify new pathophysiological mechanisms that can be targeted in isolation or in combination with existing therapeutic regimens. The epithelial-to-mesenchymal transitions (EMT) induced by hypoxia, cytokines, and growth factors involves acquisition of invasive and migratory properties by cancer cells. Epigenetic alterations of DNA methylations and/or histone modifications cause substantial transcriptomic reprogramming in cancer cells during EMT and metastasis, which can be therapeutically targeted by a thorough understanding of the mutual interactions among the epigenetic processes. Previously, the mammalian DNA methyltransferases (DNMTs) have been shown to possess redox- and Ca^++^- dependent active DNA 5mC demethylation activities in addition to the cytosine methylation activity.

**Methods:**

In this study, we have carried out experiments using a range of molecular, cellular, and genome editing approaches including cell culturing, CRISPR/Cas9-editing, si- or sh-RNA-mediated knockdown, quantitative RT-PCR, western blotting, ChIP-qPCR, Na-bisulfite sequencing, EMT and lung colonization assays in conjunction with DNA methylome and DNMT3A ChIP-Seq analyses,

**Results:**

We found that active DNA demethylation activity of DNMT3A is essential for hypoxia-induced EMT of the SW480 colon cancer cells, its global genomic DNA demethylation, and promoter DNA demethylation/transcriptional activation of EMT-associated genes including *TWIST1* and *SNAIL1*. DNMT3A also regulates hypoxia-induced HIF-1α binding to and transcriptional activation of the *TWIST1* promoter as well as genome-wide DNA demethylation and EMT of breast cancer and liver cancer cells. Mechanistic analysis supports a regulatory model where hypoxia-induced H3K36me3 mark recruits DNMT3A to demethylate CpG in the hypoxia-responsive element (HRE), thereby facilitating HIF-1α binding and activation of the promoters of EMT genes*.*

**Conclusions:**

Altogether, this study has provided the first demonstration of a physiological function of the active DNA demethylation activity of the DNMTs. Equally important, our findings have revealed a missing link between the HIF-1α pathway and the O_2_-sensing KDM pathway both of which are known to be essential for a wide set of normal and disease-associated cellular processes. Finally, the active DNA demethylation activity of DNMT3A has now emerged as a new potential target for therapeutic development to prevent EMT and metastasis of cancer cells.

**Clinical trial number:**

Not applicable.

**Supplementary Information:**

The online version contains supplementary material available at 10.1186/s11658-025-00775-x.

## Background

Establishment and maintenance of specific gene transcription profiles from eukaryotic genomes are tightly linked and required for normal development, physiological processes, and progression of diseases such as cancer [1]. Apart from the genetic factors, transcription of the eukaryotic genomes is also subjected to tight spatial and temporal regulation via several epigenetic modifications, including *C*-5 methylation of DNA cytosines (5-mC) mainly within CpG contexts [2] and post-translational modifications (PTM) of histones, such as methylation and acetylation of their *N*-terminal tails [3]. DNA methylation of the vertebrate genome is catalyzed by a family of DNA methyltransferases, or DNMTs, among which DNMT1 primarily functions to maintain existing DNA methylation patterns during replication. In contrast, DNMT3A and DNMT3B are de novo enzymes that establish the initial genome methylation patterns [4–6]. The presence of 5-mC on DNA can enhance or reduce transcription, depending on the proximal chromatin context [7]. On the other hand, active demethylation of DNA 5-mC is achieved via multiple enzymatic pathways [8–11]. In particular, the actions of the TET family of dioxygenase enzymes oxidize 5-mC and then nucleotide excision repair (NER) and base excision repair (BER) can remove the oxidized forms of 5-mC. Surprisingly, in addition to their well-known methyltransferase activity, the mammalian DNMTs have been shown to possess DNA 5-hmC dehydroxymethylation activity and Ca^2+^/redox condition-dependent 5-mC demethylation activity in vitro and in transfected cells [12–14], though the physiological roles of these activities have remained unclear.

The epithelial-mesenchymal transition (EMT) is a critical step in carcinogenesis whereby cancer cells of an epithelial nature undergo de-differentiation through a series of transcriptional events regulated by epigenetic reprogramming, which confers on cancer cells increased proliferative potential and higher invasive and metastatic abilities [15–18]. The hypoxic environment causes the generation of excessive reactive oxygen species (ROS) [19, 20] known to exert myriad effects to alter the epigenetic landscapes of cancer cells and other cellular models of hypoxia by interfering with well-known epigenetic modifications including DNA methylation, histone methylation/acetylation and non-coding RNAs [21, 22]. The transcription of a number of epithelial and mesenchymal genes is regulated by histone PTM processes, such as acetylation and methylation, with the latter involving the combined activities of histone methyltransferases [3] and lysine-specific histone demethylase (LSD) [23], as well as O_2_-sensing Jumonji C (jmjC) domain-containing histone demethylases [24–27]. Hypoxia is one of the potent inducers of EMT of cancer cells through the participation of the master regulator hypoxia-inducible factors (HIFs) that function in the transcriptional activation of a number of genes through binding to the hypoxia-response element (HRE) in their promoters [18]. Notably, ROS-mediated oxidative stress reduces the activity of jmjC domain-containing histone demethylases [28].

Hypoxia-induced binding of HIF transcription factors to the HRE sequence is greatly affected by the methylation level of the CpG motif present within it [29, 30]. Interestingly, D’Anna et al. [29] have reported that treatment of MCF-7 breast cancer cells with 5-aza-2′-deoxycytidine, an inhibitor of the DNA methylation activity of DNMTs, would expose several HREs for HIF1-*β* binding. Moreover, DNA methylation state of the promoters of EMT-related genes can be associated with their expression. For example, hypermethylation of the promoter DNA of *TWIST1*, an EMT transcription factor-encoding gene, is inversely correlated with its expression in different colon and breast cancer cell lines [31, 32]. Also, silencing of *TWIST1* in a gastric cancer cell line is associated with heavy methylation of its promoter DNA, and the gene transcription could be reactivated upon treatment with 5-aza-2′-deoxycytidine [33]. In the following, we provide the first direct evidence that one of the DNA methyltransferases, DNMT3A, is required for hypoxia-induced transcriptional activation of the EMT-associated gene *TWIST1* in cell lines of three different cancer cell types. Furthermore, in the colon cancer SW480 cells, DNMT3A appears to be recruited by the hypoxia-induced histone mark H3K36me3 to demethylate the HRE in the *TWIST1* promoter, thus allowing HIF-1α binding and transcriptional activation of the promoter to initiate the EMT process. The data presented uncovers a functional role of the DNA demethylation activity of DNMT3A in vivo, which appears to link the HIF pathway and O_2_-sensing KDM pathway for the regulation of hypoxia-induced EMT of cancer cells.

## Methods

### Cell cultures, plasmid DNA constructs, antibodies, DNA primers/oligos

The human colon cancer cell line SW480 (Cat No. CCL-228) was obtained from ATCC, USA. The breast cancer cell line MCF-7 (Cat No. HTB-22, ATCC, USA) and hepatocellular carcinoma cell line Hep G2 (Cat No. HB-8065, ATCC, USA) were kind gifts from Prof. Steve R. Roffler and Prof. Yuh-Shan Jou from the Institute of Biomedical Sciences, Academia Sinica, Taiwan, respectively. All of these cell lines were tested and found to be free of mycoplasma contamination. All the cell lines were cultured under 5% CO_2_ at 37 °C in Dulbecco’s Modified Eagle’s Medium (DMEM) (Invitrogen) supplemented with 10% fetal bovine serum (FBS) (v/v) (Biological Industries) and 1% (v/v) penicillin–streptomycin (Invitrogen). Knockdown of endogenous DNMT3A and TET1 expression was accomplished using shRNAs that targeted *DNMT3A* and *TET1* mRNA, respectively, and they were expressed from pLKO_TRC005 vector (RNAi core, Academia Sinica). The expression of endogenous TET2 and TET3 was knocked down using respective siRNAs targeting *TET2* and *TET3* mRNAs. To construct SW480 cell lines with DNMT3A-knockout, DNMT3A-C710A-knock-in, and DNMT3A-R885A-knock-in, respectively, p5w-Cas9.pBsd vector was used to express Cas9 endonuclease under the human EF1-*α* promoter, and pU6-sgRNA.Ppuro vector was used to express sgRNAs under the human U6 promoter. Wild type human *DNMT3A* cDNA and its variants encoding DNMT3A-C710A and DNMT3A-R885A were cloned into the EcoRI and NotI sites of pCI vector (Promega) to generate constructs pCI-hDNMT3A, pCI-hDNAMT3A-C710A and pCI-hDNMT3A-R885A, respectively. All the oligonucleotides including Fluorescein amidites (FAM)-labeled primers used in EMSA were from PURIGO Biotech Inc. The sequences of various DNA primers and oligos employed are listed in Table 1.

**Table 1:** Nucleotide sequences of primers

Category	Name	Sequence (5′ to 3′)
Quantitative RT-PCR	h3A-qRT-F	GCCCAAGGTCAAGGAGATTATT
	h3A-qRT-R	GAGATGCAGATGTCCTCAATGT
	GLUT1-qRT-F	TTGCAGGCTTCTCCAACTGGAC
	GLUT1-qRT-R	CTTCACTGTGCTCCTGGTTCTG
	Twist1 Homo QF	TCTCGGTCTGGAGGATGGAG
	Twist1 Homo QR	TTCTCTGGAAACAATGACATCTAGG
	Snail1 Homo QF	CCCAATCGGAAGCCTAACTACA
	Snail1 Homo QR	AGGACAGAGTCCCAGATGAGC
	Vegfa Homo QF	CTGGAGCGTGTACGTTGGT
	Vegfa Homo QR	GTTTAACTCAAGCTGCCTCGC
	Actin Homo QF	CCTGAACCCCAAGGCCAACC
	Actin Homo QR	CAGGGATAGCACAGCCTGGA
	hTET1-qRT-F	GCTGCTGTCAGGGAAATCAT
	hTET1-qRT-R	AATTGGACACCCATGAGAGC
	hTET2-qRT-F	CTTTCCTCCCTGGAGAACAGCTC
	hTET2-qRT-R	TGCTGGGACTGCTGCATGACT
	hTET3-qRT-F	GTTCCTGGAGCATGTACTTC
	hTET3-qRT-R	CTTCCTCTTTGGGATTGTCC
	hESCO1-qRT-F	ATATGTGGGCTGGAAGAAAGAA
	hESCO1-qRT-R	CAGGGCATACTTTGGGTCTT
	hMST1-qRT-F	TCAAGGATGTGCTGATTCCC
	hMST1-qRT-R	TCTGGGCCTGGTCTATGTAT
Na-Bisulfite PCR	Twist1-BS-F	TTATTTTTTTAGTTTTAGTAATTTTAAAT
	Twist1-BS-R	AAAACAACAATATCATTAACCTAAC
	SNAIL1-BS-F	GGGTTAGGTTGTTTTGTAAAAAGG
	SNAIL1-BS-R	CCACGCCCCTTTATCACCTC
	pGL3-CMV-F	TGTAGGTGTTAGAATATTTTTTTAT
	pGL3-CMV-R	TCTATAATTTATATTCAACCCATAT
	pGL3-CMV-Nest-F	GATTTTATGGGATTTTTTTATTTGGTAGT
	pGL3-CMV-Nest-R	ACTAAACCAACTCTACTTATATAAACC
ChIP-qPCR	TWIST1 Homo ChIP QF	CGGGGGAGGGGGACTGGAAAGC
	TWIST1 Homo ChIP QR	AGGCCTCCTGGAAACGGTGCCG
	TWIST1 HomoD ChIP QF	TACTCCAGCGCGGTGCACAAAACT
	TWIST1 HomoD ChIP QR	AACGAAGAGCCCCAAAGAGGGTGT
	hSNAIL1 ChIP-F	GAAGGAACGGGTGCTCTTG
	hSNAIL1 ChIP-R	ACATCACTGGGGAGGAAGC
	hESCO1 ChIP-F	CCTCTCGAGTAGCTGGGATTA
	hESCO1 ChIP-R	CATGGCCAACATGGTGAAAC
	hMST1 ChIP-F	TCCCAAAGTGCTGGGATTAC
	hMST1 ChIP-F	CAGGAGGATTGCTTGAGGTTAG
	HRE_9656652 ChIP-F	GCCTGTAGTCCCAGCTACT
	HRE_9656652 ChIP-R	AGTGCAGTGGCATGATCTC
	HRE_123098647 ChIP-F	CTGCCTGTTCTGTTTACACTTATG
	HRE_123098647 ChIP-R	GATATGAAAGCACCTATCGCATTG
DNMT3A shRNA related	sh-3A-1	CCGGCTCTTCTTTGAGTTCTA
	sh3A-2	CCCAAGGTCAAGGAGATTATT
TET knockdown related	sh-TET1	GTAGACCATCACTGTTCGAC
	si-TET2	GCUCUGAACGGUAUUUAAA
	si-TET3	CCGGCAGUUUGAGGCUGAAUUUGGA
DNMT3A-KO related	hDNMT3A-KO-sg-1	GACGATGGAGAGGTCATTGC
	hDNMT3A-KO-sg-2	GGCCGTGAGGTGCTCATGTG
	hDNMT3A-KO-sg-3	GAATGCTGTGGAAGAAAACC
DNMT3A-C710A knock-in related	h3A-C710A KIN-sg	GACGATGGAGAGGTCATTGC
	h3A-C710A KIN-donor	ATCCAGGAGTGGGGCCCATTCGATCTGGTGATTGGGGGCAGTCC TGCTAATGACCTCTCCATCGTCAACCCTGCTCGCAAGGCCTCTACG
DNMT3A-R885A knock-in related	h3A-R885A KIN-sg	GCCAACATGAGCCGCTTGGCG
	h3A-R885A KIN-donor	GGTATTTGGTTTCCCAGTCCACTATACTGACGTCTCCAACATGAGCCGCT TGGCGGCCCAGAGACTGCTGGGCCGGTCATGG

Na-Bisulfite PCR: Sodium-Bisulfite PCR; ChIP-qPCR: Chromatin Immunoprecipitation-quantitative PCR; shRNA: Short hairpin RNA; DNMT3A-KO: DNA methyltransferase 3A-knockout; TET: Ten-eleven translocation family proteins

Anti-DNMT3A (Cat No. NB120-13888, RRID: AB_789607) and anti-DNMT3B (Cat No. NB100-56514, RRID: AB_838139)) were purchased from Novus Biologicals. Anti-DNMT1 (Cat No. ab19905, RRID: AB_731983) was purchased from Abcam. Anti-TET1 (Cat No. NBP3-11863) was procured from Novus Biologicals, and anti-TET2 (Cat No. 21207-1-1AP) came from Proteintech. Anti-TET3 (anti-Ppo3C) was a generous gift from Tianpeng Gu and Guo-Liang Xu at the Chinese Academy of Sciences, Shanghai. Anti-HIF-1α was purchased from Proteintech (Cat No. 20960-1-AP) and Novus Biologicals (Cat No. NB100-105). The anti-TDG (Cat No. 13370-1-AP) came from Proteintech. The anti-H3K9/K18Ac (Cat No. 07-593) and anti-H3K9me3 (Cat No.07–442, RRID: AB_310620) came from Sigma Aldrich, and anti-H3K27me3 (Cat No. ab6002, RRID: AB_305237) and anti-H3K36me3 (Cat No. ab9050, RRID: AB_306966) came from Abcam. The anti-*α*-Tubulin (Cat No. T-5168) was purchased from Sigma Aldrich.

### shRNA or siRNA-mediated knockdown (KD) of *DNMT3A* and *TET* mRNAs and CRISPR/Cas9-mediated knockout (KO) of DNMT3A expression in cancer cells

To knockdown endogenous DNMT3A expression in SW480 cells, we transfected early passage cells with pLKO_TRC005 plasmid constructs encoding shDNMT3As or shTET1, respectively (Table 1) using Lipofectamine 2000 reagent (Thermo Fischer Scientific). Two different shRNAs were used to target *DNMT3A* in SW480 cells (Fig. S1B). At 17 h post-transfection, fresh medium containing 5 μg/mL of puromycin (Invitrogen) was used for cell selection for 48 h before further experimentation. We used sh-3A-1 in all experiments to knock down *DNMT3A* mRNA expression in SW480 cells. The sh-3A-1 was also used to knock down endogenous *DNMT3A* expression in MCF-7 and Hep G2 cell lines.

To generate CRISPR/Cas9-mediated knockout (KO) of the endogenous DNMT3A, three sgRNAs (Table 1) targeting human *DNMT3A* gene were cloned into pLAS-CRISPR-NG expression vector (provided by the C6 RNAi core facility, Academia Sinica, Taiwan), which also expresses eSpCas9. The pLAS-CRISPR-NG plasmid encoding the above sgRNAs and eSpCas9 was then transfected into SW480 cells by using Lipofectamine 3000 reagent (Thermo Fischer Scientific). Puromycin was added 17 h later, and the transfected cells were grown for another 72 h. Subsequently, the puromycin-selected cells were subjected to single-cell clonal isolation and continually cultured until the formations of visible colonies. Individual visible colonies were then expanded and screened for the expression of *DNMT3A* mRNA and DNMT3A protein by RT-qPCR and western blotting, respectively (Fig. S1B).

Note that human *DNMT3A* gene encodes seven different transcripts which generate five different protein isoforms (a–e) (https://www.ncbi.nlm.nih.gov/gene). While the longest isoform (a) contains 912 amino acids, use of a downstream promoter would encode transcripts that generate DNMT3Ab (723 amino acids) and DNMT3Ad (760 amino acids). Another two isoforms, DNMT3Ac (166 amino acids) and DNMT3Ae (689 amino acids) have also been reported. Either the shRNAs used in the knock-down experiments or the sgRNAs used in the knock-out experiments were designed to target the expression of all five DNMT3A isoforms. To generate homozygous knock-in mutations C710A and R885A, pLAS-CRISPR-NG expression vector was used to clone sgRNAs, targeting C710A and R885A of DNMT3A (Table 1). The early passage SW480 cells were then co-transfected with either pLAS-CRISPR-NG expressing sgRNA targeting C710A together with C710A-specific single-stranded donor template or pLAS-CRISPR-NG expressing sgRNA targeting R885A together with R885A-specific single-stranded donor template (Table 1) using Lipofectamine 3000 reagent (Thermo Fischer Scientific). Puromycin was added 17 h later, and the transfected cells were grown for another 72 h. Subsequently, the puromycin-selected cells were subjected to single-cell clonal isolation followed by sequencing of genomic DNAs harvested from the knock-in clones (Fig. S2).

The choice of KO cell lines over KD cells for analysis in most of our experiments was because of the complete absence of DNMT3A expression in the KO cells. Furthermore, while both SW480-3A-KO-1 and SW480-3A-KO-2 exhibited suppression of the hypoxia-induced EMT phenotypes, we randomly chose SW480-3A-KO-1 line for our study. Moreover, we randomly chose SW480-3A-C710A-1 and SW480-3A-R885A-1 clones for the analysis of the effects of the C710A and R885A mutations.

### Hypoxia treatment of parental and genome-edited cancer cell lines

For hypoxia treatment, the parental, pooled SW480 cells and different cell lines (SW480-3A-KO-1/SW480-3A-C710A-1/SW480-3A-R885A-1, MCF-7 and Hep G2) were transferred from a normoxic incubator (5% CO_2_ and 20% O_2_) to a hypoxic incubator (5% CO_2_ and 1% O_2_) for 24 h. Increased levels of HIF-1α, as assayed by western blotting, were used to assess the induction of hypoxia.

### Na-bisulfite sequencing of *TWIST1* promoter DNA

Genomic DNAs were isolated from parental and genetically engineered SW480 cell lines grown under normoxia or hypoxia. We subjected 500 ng of genomic DNA from each of the cell lines to Na-bisulfite conversion using a EZ DNA methylation Gold Kit (Zymo Research, USA). The bisulfite-converted DNAs were subjected to PCR amplification using appropriate primer pairs (Table 1). The amplicons were then cloned into pJET 1.2/blunt vector and the resulting individual plasmid clones were sequenced using vector-specific DNA sequencing primers.

Note that Na-bisulfite sequencing could not differentiate between C and hmC [34]. For detecting the presence of 5-hmC in the *TWIST1* promoter, DNA glucosylation and restriction endonuclease digestions were performed using the Epimark 5-hmC and 5-mC analysis Kit (NEB, USA) following the manufacturer instructions [35]. The result of this assay showed absence of 5-hmC in the proximal promoter of *TWIST1* before or after hypoxia (data not shown).

### Analysis of the methylomes of SW480 cells under normoxia (N) and hypoxia (H) conditions

The methylomes were analyzed by whole genome bisulfite sequencing (WGBS). Genomic DNAs from SW480 cells without or with hypoxia treatment were fragmented to an average size of 300 bp with a Covaris M220 sonicator using a specific program. The sheared DNA was purified and size selected using Ampure beads. Bisulfite conversion of DNA was then conducted by using the EZ DNA Methylation-Gold kit (Zymo Research). The bisulfite-converted DNA was immediately processed for generating WGBS libraries using the Accel-NGS Methyl-Seq DNA library kit (Swift Biosciences). The libraries were quantified using KAPA Library Quantification Kits for Illumina Platforms (Kapa Biosystems) and then sequenced on an Illumina NextSeq500 and HiSeq X Ten sequencer as 150-bp paired-end reads.

The WGBS raw data were analyzed by using default parameters via BatMeth2 [36]. The data processing including the alignment with the human genome (GRCh38.p13; GCF_000001405.39) and calculation of DNA methylation levels of CpG sites was accomplished using the function “Calmeth”. The identification of differentially methylated cytosines (DMC) and differentially methylated regions (DMR) was achieved using the function “batDMR.” The HRE regions with the sequence element 5′-(G/A)CGTG-3′/3′-CACG(C/T)-5′ were identified and then filtered by the differentially methylation levels (methdiff > 0.2) of CpG sites to identify sites undergoing demethylation upon hypoxia treatment.

The methylation levels of the CpGs including the ones present in HRE sequences in the *TWIST1* and *SNAIL1* promoter regions were measured under normoxia and hypoxia conditions in wild type and DNMT3A-ablated SW480 cells.

### Analysis of genomic 5-mC content

To measure the genomic C and 5-mC contents, 1 µg of the genomic DNA harvested from the parental-and DNMT3A-depleted SW480, MCF-7 and Hep G2 cells under normoxia and hypoxia was subjected to degradation up to the individual nucleoside components using 5 units of DNA Degradase Plus enzyme cocktail (Zymo Research) in 25 μL volume at 37 °C for 2.5 h, followed by heat inactivation of the enzyme mixture at 70 °C for 20 min. The resulting degraded products were filtered through NANOSEP 3 K OMEGA Acrodisc syringe filters (PALL Corp.) and the C or 5-mC nucleosides were detected by LC–ESI–MS/MS on a Velos Pro mass spectrometer (Thermo Fisher Scientific, San Jose, CA) equipped with standard ESI ion source, an Agilent 1100 Series binary high-performance liquid chromatography pump (Agilent Technologies, Palo Alto, CA), and a Famos autosampler (LC Packings, San Francisco, CA) using XBridge BEH C18 Column (1.0 mm X 150 mm, 3.5 um, Waters, Milford, MA).

### ChIP-qPCR assay

The ChIP assay followed the standard procedure [37] with some modifications. Parental or genetically engineered SW480 cell lines grown under normoxia or hypoxia were fixed with 1% formaldehyde for 10 min at room temperature. The fixed cells were sonicated (BRANSON sonifier 150) to shear the chromatin. Then, 70 μg of the sheared chromatin was pre-cleared for 2 h using Protein *A*- or Protein *G*-sepharose beads (GE Healthcare) and subjected to immunoprecipitation overnight in the presence of different antibodies or normal rabbit immunoglobulin G (NrIgG). The immune-complexes were captured using either Protein *A*- or Protein *G*-sepharose beads for 4 h, followed by sequential washes of the beads with buffer. The captured DNAs were retrieved and subjected to quantitative PCR amplification (qPCR) using primer sets covering the proximal and distal regions of the *TWIST1* and *SNAIL1* promoters.

### ChIP-Seq analysis of DNMT3A-binding on the SW480 genome and in the *TWIST1* promoter

For ChIP-Seq analysis, we immunoprecipitated fragmented-chromatin from SW480 cells under normoxia and hypoxia conditions, respectively using anti-DNMT3A antibody as described above. The DNA libraries were prepared using KAPA HyperPrep library preparation kit (Roche) following standard protocols. Samples with different dual-indexed barcodes were pooled at equal molar ratio and sequenced on NextSeq500 system according to the standard protocol from illumina with 75-bp single-end reads.

To analyze the ChIP-Seq data, we initially utilized bowtie2 [38] for read mapping to the human genome (GRCh38.p13; GCF_000001405.39). Subsequently, we employed “Sambamba” to filter out the read alignments with multiple hits [39]. For peak calling, we utilized “MACS2” to identify both narrow and broad peaks [40]. We retained only those peaks having above fivefold enrichment and a *q*-value above 0.05.

For examination of DNMT3A-binding in the *TWIST1* promoter region, we identified consensus peaks present in both the replicates for normoxia and hypoxia conditions.

### Electrophoretic mobility shift assay (EMSA)

For EMSA, nuclear extracts prepared from normoxic or hypoxic SW480 cells were incubated with 5 nM of non-methylated (FAM-5′-CCTCCTCACGTCAGGCCA-3′/3′-TGGCCTGACGTGAGGAGG-5′) or methylated (FAM-5′-CCTCCTCA^m^CGTCAGGCCA-3′/3′-TGGCCTGA^m^CGTGAGGAGG-5′) *TWIST1* HRE- containing DNA oligo probe in the binding buffer (10 mM Trist-HCl, pH 7.5, 1 mM MgCl_2_, 0.5 mM EDTA, 50 mM NaCl, 0.5 mM DTT, 10% Glycerol (v/v), 0.5 mg/mL poly(dI-dC).poly(dI-dC)) for 20 min, followed by separation of the protein-DNA oligo complexes on 5% native polyacrylamide gel. For competition assay, excess (50× or 100×) unlabeled and non-methylated DNA oligo was included during the incubation and before gel loading. For detection of the super-shifted HIF-1α-DNA oligo complex, the binding reaction was followed by further incubation with normal rabbit IgG or anti- HIF-1α antibody for 1 h at 4 °C before gel loading. The gel was visualized in Typhoon FLA 9000 (GE healthcare Life Science) with use of 600 V PMT voltage and 100 μm pixel size.

### Growth, colony-formation, wound healing, and lung-colonization assays

For the cell growth assay, 10^4^ SW480 cells/well were seeded on a 96-well plate in DMEM medium with 10% FBS and 1% penicillin–streptomycin, and the cell growth was monitored by measuring the absorbance at 600 nm using AlamarBlue assay reagent (Thermo Fischer Scientific) at 24, 48 and 72 h time-points. For the colony-formation assay, the SW480, MCF-7 and Hep G2 cells were seeded in 10-cm culture dishes (2000 cells/dish) and grown for 2 weeks to obtain distinct colonies. Subsequently, the colonies were stained with Giemsa stain and counted manually. For the wound-healing assay, the SW480, MCF-7 and Hep G2 cells were grown to full confluency on 6-cm culture dishes for 24 h. The confluent monolayer was then scratched using a sterile 0.2 mL pipette tip to generate two parallel lines with an enclosed empty region. After 24 h, the migrating cells from the edge of the scratch were imaged using an inverted fluorescence microscope (Olympus). Relative cell migration was analyzed using ImageJ software (RRID: SCR_003070).

For the lung colonization assay, 10^6^ pLKO.1- or pLKO.1-sh3A-1-transfected and normoxia-or hypoxia-treated SW480 cells were injected into 8-week-old male nude mice through the tail vein. The mice were then sacrificed 6 week later to analyze the number of tumor foci in their lungs. For each group, 4–5 mice were injected and no exclusion criteria were imposed during counting of the tumor foci. While injecting the cells through tail vein, the mice were allocated through simple randomization and no blinding was carried out. The sample size of the animals was determined based on the minimum error rate. All animals used in this study were maintained in a specific pathogen-free (SPF) environment under standard laboratory conditions and handled following the guidelines of the Institutional Animal Care and Use Committee of Academia Sinica (AS IACUC).

### Western blotting analysis of whole cell extracts

Whole cell extracts from cells grown under normoxic or hypoxic conditions were prepared, resolved on 8% or 15% SDS-PAGE, and then probed with antibodies detecting HIF-1α, DNMT3A, DNMT3B, TET1, TET2, TET3, TDG, *α*-tubulin proteins or the H3K36me3 and H3K27me3 histone modification marks, respectively.

### Luciferase reporter assay measuring the methylation and demethylation activity of human DNMT3A

HEK 293 cells were co-transfected with empty pCI vector, pGL3-CMV (for the methylation assay), or methylated pGL3 (for the demethylation assay) together with the expression plasmids pCI-hDNMT3A, pCI-hDNMT3A-C710A or pCI-hDNMT3A-R885A for 48 h, followed by measurement of the Lucia and firefly luciferase activities. The 1.0 luciferase activities in the methylation and demethylation assays were computed by normalizing the firefly luciferase readings with the corresponding Lucia luciferase readings.

### Statistical analysis

All data were derived from at least three independent experiments and they are represented as mean ± standard deviation (SD). Statistical significance was determined by unpaired *t* test. We did not use any statistical method to perform sample size calculation. Rather, the sample sizes were determined on the basis of the minimum error rate.

## Results

### Requirement of DNMT3A for hypoxia-induced EMT of colon cancer cells

**Fig. 1:**
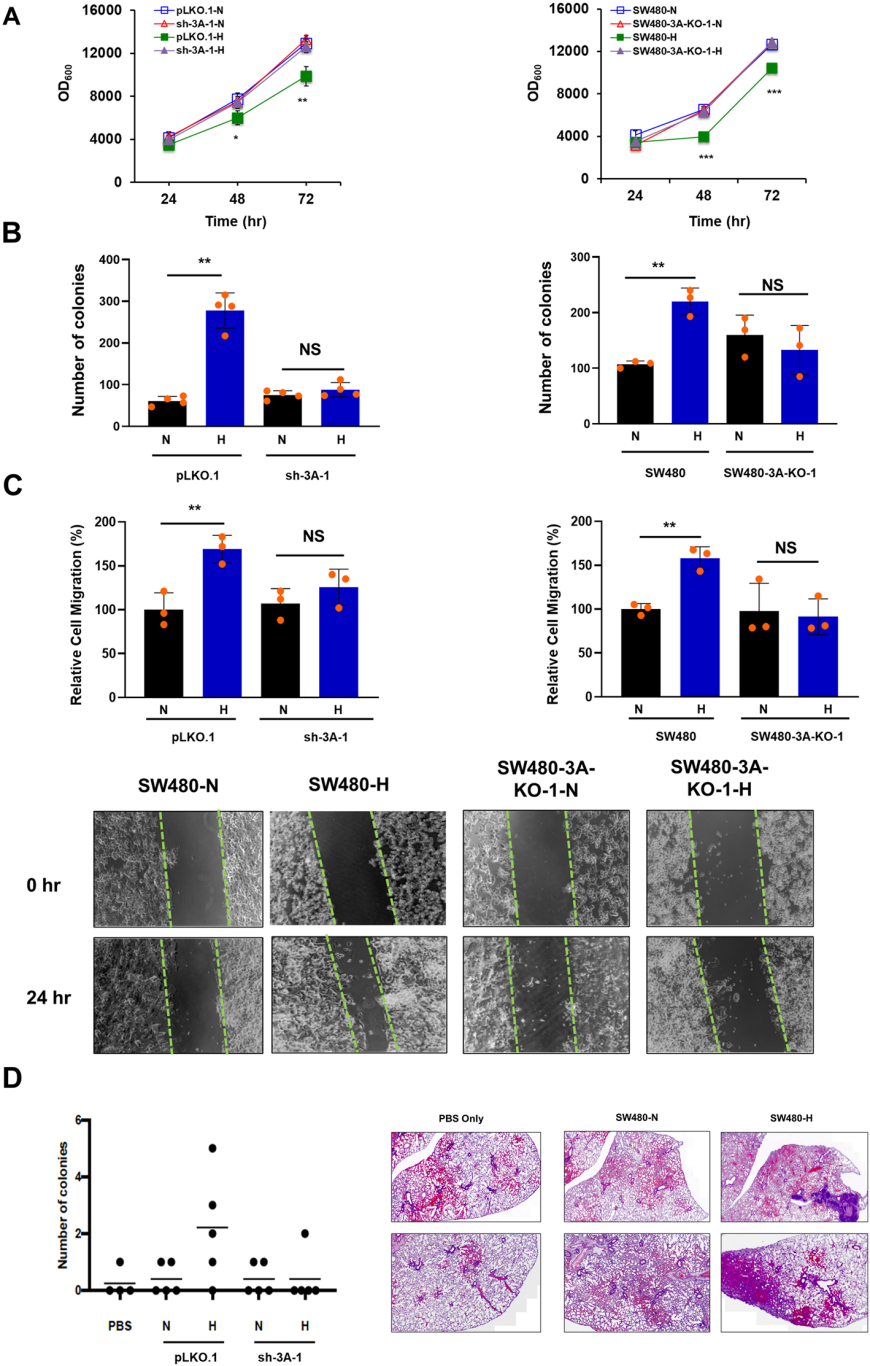
Effect of DNMT3A depletion on EMT-associated cellular phenotypes of hypoxia-treated SW480 cells. **a** Growth kinetics under normoxia and hypoxia of SW480 cells upon shRNA-mediated depletion (left panel) or CRISPR/Cas9-mediated ablation (right panel) of DNMT3A expression over a 72-h period after seeding the cells. Each point on the line plot represents mean ± SD of absorbance measured at 600 nm. **b** Colony formation assay of SW480 cells under normoxia (N) or hypoxia (H) upon shRNA-mediated depletion (left panel) or CRISPR/Cas9-mediated ablation (right panel) of DNMT3A expression. Each histobar represents mean ± SD of the number of colonies that had formed 2 weeks after cell seeding. **c** Relative cell migration (%), as determined by wound-healing assay, for SW480 cells upon shRNA-mediated depletion (top left panel) or CRISPR/Cas9-mediated ablation (top right panel) of DNMT3A expression. Each histobar represents mean ± SD of migrating cells relative to the value for wild-type SW480 cells under normoxia condition. The bottom panels show the representative images from wound healing assay of migrating wild type SW480 cells and SW480-3A-KO-1 cells under normoxia (N) and hypoxia (H) conditions. **d** Lung colonization assay. Numbers of colonies on the lungs of mice injected into the tail vein with SW480 cells with or without endogenous DNMT3A knockdown by shRNA and grown under normoxia (N) or hypoxia (H) were counted and plotted (left panel). The number of colonies for each mouse is indicated by black dots, and average numbers for group of mice are indicated by the horizontal bars. Shown in the right-side panels are representative histological images of the H&E-stained lung sections derived from mice sacrificed after 35 days following tail-vein injection of PBS only (PBS), SW480 cells grown under normoxia condition (SW480-*N*), and SW480 cells grown under hypoxia condition (SW480-H), respectively. In A, B, and C, mean ± SD was derived from three or more independent biological replicates each with three technical repeats. *, *p* < 0.05; **, *p* < 0.01; ***, *p* < 0.005; NS, not significant

We first investigated whether and how DNMT3A regulates hypoxia-induced EMT, using the human colon cancer cell line SW480 [41] as a cellular model. Expression of endogenous DNMT3A was depleted by either shRNA-mediated knockdown or CRISPR/Cas9-mediated ablation to generate sh-3A or SW480-3A-KO cell populations, respectively. Both parental and DNMT3A-depleted cells were then subjected to either normoxic or hypoxic conditions for 24 h, before undergoing further molecular and cellular analysis (Fig. S1A). We found that a 70–80% reduction of DNMT3A in sh-3A cell pools or absence of DNMT3A in SW480-3A-KO cell lines (Fig. S1B) did not affect the expression levels of TET1, TET2, TET3, and TDG under normoxia or hypoxia, as exemplified in the Fig. S1C (Upper panels). Also, CRISPR/Cas9-mediated ablation of DNMT3A in SW480 cells did not alter the DNMT3B or DNMT1 expression levels as shown in SW480-3A-KO-1 and SW480-3A-KO-2 cell lines when compared with parental SW480 cells under normoxia and hypoxia conditions (bottom panels of Fig. S1C). As expected, in all cases, hypoxia induced a robust increase of the hypoxia-inducible factor HIF-1α as evidenced by the histograms of HIF-1α (Fig. S1D). However, EMT of SW480 cells were prohibited upon depletion of DNMT3A. In particular, known properties of EMT, i.e., reduced cell growth (Fig. 1a), increased colony formation ability (Fig. 1b), enhanced migration capability in wound-healing assay (Fig. 1c), and elevated metastasis in mice (Fig. 1d) all disappeared in SW480 cells subjected to knockdown or knockout of DNMT3A expression. To control for possible off-target effects associated with CRISPR/Cas9-mediated genome editing, we subjected SW480 clones in which DNMT3A was not successfully deleted by editing to the above mentioned assays and failed to find significant changes in hypoxia-induced EMT-associated phenotypes in comparison to the parental SW480 cells (data not shown). Noteworthy to mention here is that we observed only a minor effect of DNMT3B ablation on the proliferation of SW480 cells under hypoxia condition in comparison to normoxia (data not shown).

These data together indicate that DNMT3A is an essential positive regulator of hypoxia-induced EMT in colon cancer cells.

### Requirement of DNMT3A for hypoxia-induced transcription of EMT genes and hypomethylation of the *TWIST1* promoter DNA

**Fig. 2:**
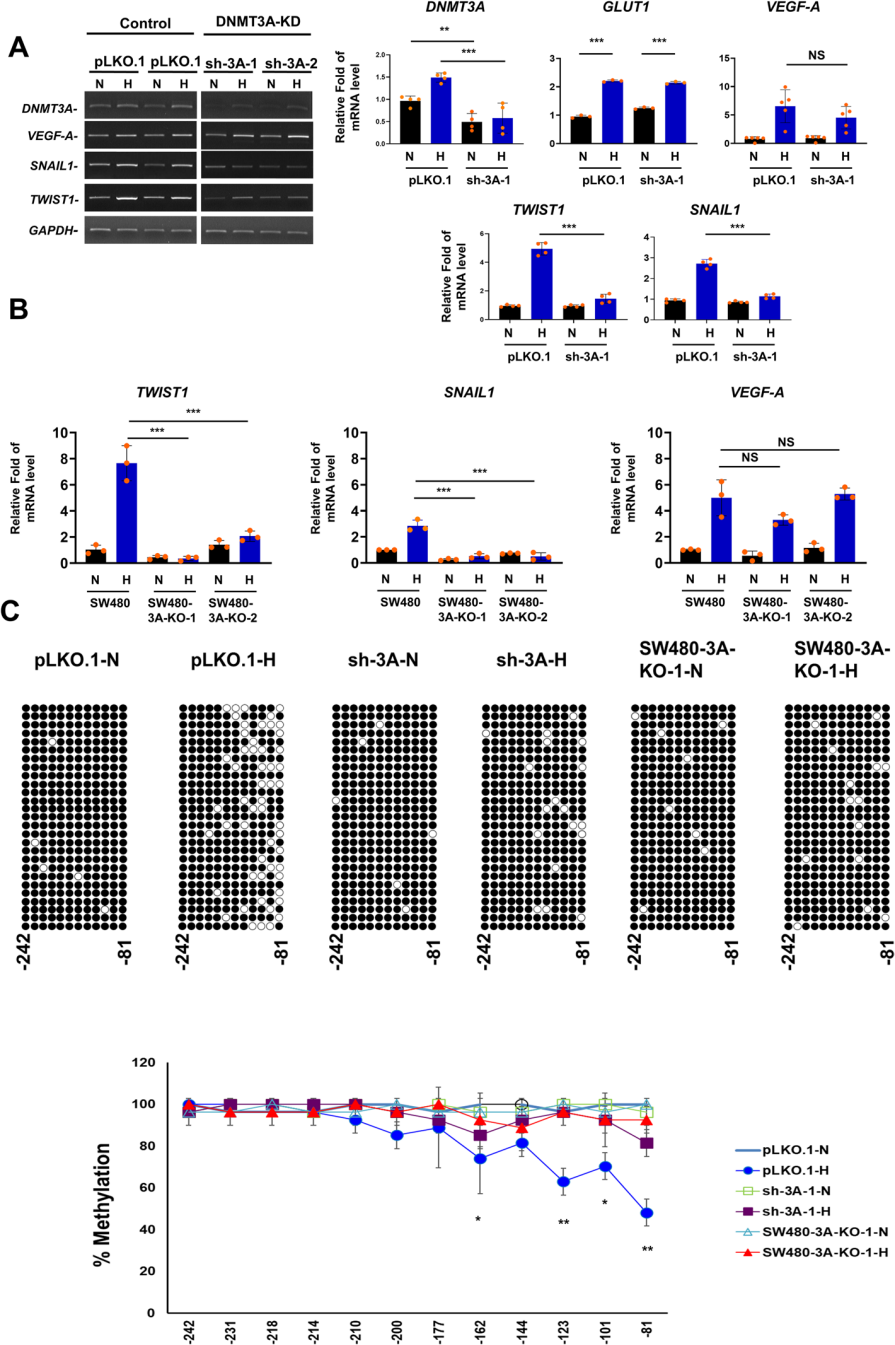
Effect of depleting DNMT3A on the expression of EMT-associated genes and CpG methylation of the *TWIST1* promoter. **a** and **b** Total RNAs harvested from SW480 cells without (control) or with depletion of DNMT3A by shRNA-mediated knockdown (**a**) or CRISPR/Cas9-mediated ablation (**b**) were subjected to quantitative-RT-PCR (both in (**a**) and (**b**)) assay. Primers specific for *DNMT3A*, *GLUT-1*, *VEGF-A*, *SNAIL1*, *TWIST1* and *GAPDH* were used in (A and B). Histobars represent mean ± SD from three or four independent biological experiments each with three technical repeats. ** and *** represent *p* < 0.01 and *p* < 0.005, respectively; NS, not significant. **c** Na-bisulfite DNA sequencing analysis of the *TWIST1* promoter region (positions −81 to −242) of parental SW480 cells compared with SW480 cells in which DNMT3A was depleted by shRNA-mediated knockdown or CRISPR/Cas9-mediated ablation. The cells were grown under normoxia (N) or hypoxia (H) condition. The clone diagram (upper panels) and bar diagram (lower panel) indicate the methylation levels of the individual CpG sites. For each of the sets, a total of 27 clones was harvested from three independent experiments. The level of methylation at each of the CpG sites is expressed as mean percentage methylation ± SD from three independent experiments. * and ** represent *p* < 0.05 and *p* < 0.01, respectively, based on Students *t* test

To understand the molecular basis underlying positive regulation of EMT by DNMT3A, we first examined the expression of four known EMT-associated genes—namely *VEGF-A*, *GLUT-1*, *SNAIL1* and *TWIST1* [42]—in parental and sh-3A/SW480-3A-KO cell populations by quantitative RT-qPCR analysis. As expected, all four genes exhibited elevated mRNA expression upon 24 h hypoxia treatment when compared with cells under normoxia. Significantly, though the *DNMT3A* mRNA level also increased under hypoxia (Fig. 2a), reduction or complete inhibition of endogenous DNMT3A expression abrogated the hypoxia-induced elevation in mRNA levels of *SNAIL1* and *TWIST1*, but not *VEGF-A* or *GLUT1* (Fig. 2a, b). Noteworthy to mention here that the expression of *SNAIL1* but not *TWIST1* mRNA under normoxia condition decreased significantly upon DNMT3A knockout that might be attributed to secondary effects associated with complete ablation of DNMT3A. In contrast, sh- or si-RNA mediated individual depletion of *TET2* or *TET3* mRNAs had no effect on the hypoxia-induced transcriptional activation of *TWIST1* gene in SW480 cells while the *TET1* mRNA depletion appeared to exert a minor inhibitory effect on this activation process (Fig. S3A). These data together indicate that hypoxia-driven transcriptional induction of certain EMT-associated genes such as *TWIST1* and *SNAIL1* rely mainly on the action by DNMT3A, directly or indirectly. Again, DNMT3B ablation showed only a minor effect on the hypoxia-induced transcriptional activation of *TWIST1* gene in SW480 cells (data not shown).

Next, we examined the DNA methylation profile of the *TWIST1* promoter region under normoxia and hypoxia by Na-bisulfite DNA sequencing (Fig. 2c). In particular, we targeted the upstream proximal promoter region of *TWIST1* (from positions −242 to −81), which encompassed a hypoxia-response-element (HRE) known to function in activation of hypoxia-inducible genes via binding with HIF-1α [43]. We found that all CpG sites within the *TWIST1* promoter of normoxic SW480 cells were almost completely methylated, but hypoxia led to significant demethylation of the promoter including CpG sites located at positions −162, −123, −101, and −81 (compare open and closed circles, Fig. 2c). However, when we depleted endogenous DNMT3A from SW480 cells, hypoxia-induced *TWIST1* promoter demethylation was no longer observed (compare open and closed square and triangle symbols, Fig. 2c). Similar result was observed for the *SNAIL1* promoter (data not shown). Consistent with the finding shown in Fig. S3A, knockdown of *TET1* or *TET2* mRNAs did not affect the hypoxia-induced DNA demethylation of the *TWIST1* promoter in SW480 cells (Fig. S3B). Significantly, the hypoxia condition also induced genomic DNA demethylation of SW480 cells, but it was not observed upon DNMT3A depletion (top panels, Fig. S4). Reduction of the endogenous expressions of *TET1* or *TET2*, on the other hand did not affect this hypoxia-induced global demethylation of the SW480 genome (Fig. S3C). The above data together demonstrate that the SW480 genome undergoes global as well as local 5-mC demethylation at the *TWIST1* proximal promoter region under the O_2_-starvation condition during EMT, and that DNMT3A plays a major role, either directly or indirectly, in the regulation of this demethylation process.

### EMT-induced HIF-1α binding, DNMT3A binding, and histone PTM changes in the *TWIST1* promoter

**Fig. 3:**
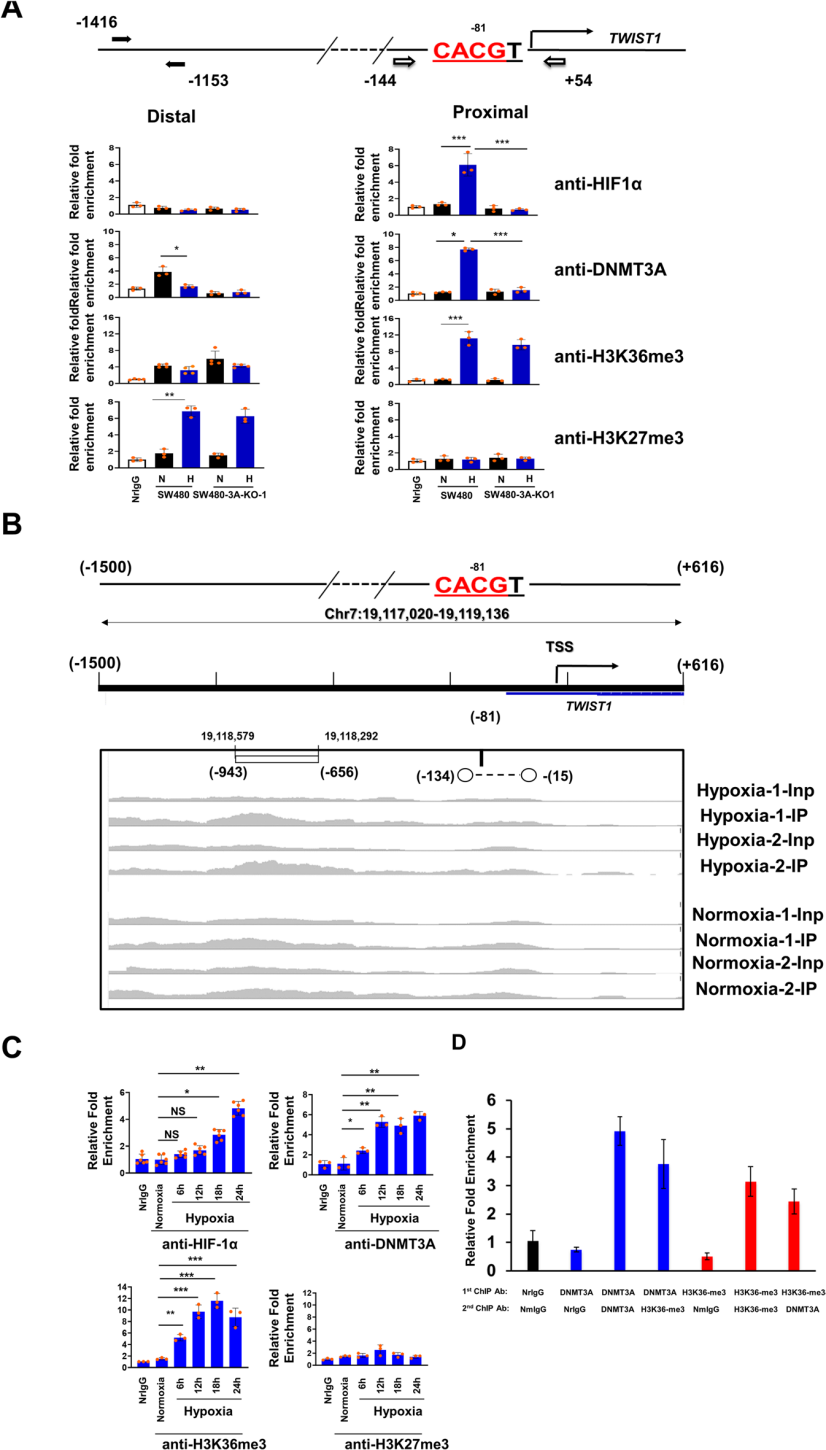
Enrichment analysis of HIF-1α binding, DNMT3A binding, and H3K36me3/H3K27me3 histone marks in the *TWIST1* promoter. **a** Top, schematic map of the *TWIST1* promoter region. Bottom panels, patterns of factor-binding or histone mark enrichment in the distal (left histographs) and proximal (right histographs) regions of the *TWIST1* promoter in wild type SW480 and SW480-3A-KO-1 cells grown under normoxia (N) and hypoxia (H) conditions, as assayed by ChIP-qPCR. Note the disappearance of hypoxia-induced HIF-1α-binding and DNMT3A-binding, but not H3K36me3 enrichment, in SW480 cells upon knockout of DNMT3A in the proximal promoter region of *TWIST1*. **b** Mapping of DNMT3A-binding in the *TWIST1* promoter of SW480 cells under normoxia and hypoxia conditions by ChIP-Seq. The DNMT3A pull-down samples are indicated as Normoxia-1-IP/Normoxia-2-IP or Hypoxia-1-IP/Hypoxia-2-IP whereas the corresponding inputs are designated as Normoxia-1-Inp/Normoxia-2-Inp or Hypoxia-1-Inp/Hypoxia-2-Inp. The consensus region (−943 to −656) for hypoxia-induced DNMT3A-binding (the horizontal bar) is derived from analysis of the replicated ChIP-Seq data. Note that in one of the replicate samples (Hypoxia-1-IP), significant enrichment of sequencing reads compared with the input (Hypoxia-1-Inp), from −134 to −15, was also obtained around the region of hypoxia-induced DNMT3A-binding as mapped by the ChIP-qPCR assay in Fig. 3a above. **c** Histograms showing the kinetics of factor-binding and histone mark enrichment in the proximal promoter region of *TWIST1* gene, as compared by ChIP-qPCR, during the hypoxia treatment (6 h, 12 h, 18 h, and 24 h) of SW480 cells. In both (A) and (C), ChIP DNA enrichment is expressed as fold-change relative to enrichment in NrIgG pulldown samples, and expressed as mean ± SD of three or more independent biological replicates each having three technical repeats. *, ** and *** represent *p* < 0.05, *p* < 0.01 and *p* < 0.005, respectively, by Students *t* test. NS, not significant. (**d**) Sequential ChIP experiment involving pulldown of the proximal *TWIST1* promoter region in parental SW480 cells subjected to hypoxia treatment for 24 h using anti-DNMT3A and anti-H3K36me3 antibodies as the first ChIP antibody, followed by the same or another antibody as the second ChIP antibody

In view of the DNMT3A-induced EMT of SW480 cells accompanied by demethylation of the HRE-containing *TWIST1* promoter (Figs. 1 and 2), we examined the in vivo chromatin binding by HIF-1α and DNMT3A using the ChIP (chromatin immunoprecipitation)-qPCR approach. As shown in Fig. 3a, the hypoxia induced binding of HIF-1α to the proximal region (−144 to + 54), but not distal region (−1153 to −1416), of the *TWIST1* promoter (top row of panels, Fig. 3a). This outcome was most likely due to recognition of the demethylated CpG (−81) in the HRE (CACGT) by HIF-1α upon hypoxia treatment, which was diminished upon DNMT3A depletion (Fig. 3a). Indeed, similar to the previous studies conducted in clear-cell renal cell carcinoma and breast cancer cell lines [29, 30], HIF-1α binding to the HRE of the *TWIST1* proximal promoter was also inhibited by CpG methylations of the element as demonstrated by EMSA (Fig. S5A). It should be noted here that the ChIP-PCR (Fig. 3a) and western blotting data (Fig. S1D, left panels) indicated that DNMTA3A mainly affected binding of HIF-1α at the EMT gene promoters, but not its stability.

To examine whether hypoxia-induced demethylation of the HREs on the SW480 genome is necessarily accompanied with binding of HIF-1α at the HRE (−81 position) of *TWIST1* promoter, we carried out whole-genome bisulfite sequencing (WGBS) of SW480 cells under normoxia and hypoxia conditions. Altogether, we retrieved 1,309,227 (82.4%) HRE-CpGs having a coverage equal to or more than 5 out of 1,588,027 HRE-CpGs in the reference genome (GRCh38). As shown in Table 2, 1,83,255 HRE-CpGs including the one present at -81 position of the proximal promoter of *TWIST1* gene underwent demethylation after hypoxia treatment. From the WGBS dataset, we selected four HRE-CpGs that underwent demethylation during the hypoxia treatment (Table 3) and subjected them to analysis of HIF-1α binding by ChIP-qPCR. Two of these HRE-CpGs, namely chr18:21601384 and chr3:49905192, resided within the 5 kb- upstream regions of the TSSs of the hypoxia-activated genes *ESCO1* and *MST1R*, respectively. The other two HRE-CpGs, namely chr18:9656652 and chr10:123098647, were located 52 kb and 37 kb, respectively, upstream of TSS of two genes. Interestingly, similar to the *TWIST1* promoter, binding of HIF-1α around the HRE-CpGs chr18:21601384 and chr3:49905192 was detected upon hypoxia treatment of SW480 cells (Fig. S6A) and this was accompanied with transcriptional activation of the respective downstream genes under hypoxia (Fig. S6A). On the other hand, however, no HIF-1α binding was found around the other two HRE-CpGs that were located far away from the transcription start sites of genes (Fig. S6B), even though these HRE-CpGs also underwent demethylation in hypoxia-treated SW480 cells (Table 3). This latter result indicates that hypoxia-induced 5mC demethylation of HRE-CpGs does not always lead to HIF-1α binding.

**Table 2:** Average methylation level of HRE-CpG (−81) and other CpGs in the proximal promoter region of *TWIST1* gene as determined by whole genome bisulfite sequencing

Position relative to TSS	Average methylation level (From WGBS)	
	*N* (%)	*H* (%)
−81	86	70
−101	85	69
−123	92	87
−144	79	77
−162	69	65
−177	80	90
−200	71	83
−210	77	80
−214	95	94
−218	92	92
−231	90	94
−242	78	100

HRE: Hypoxia-response element; TSS: Transcription start site; WGBS: Whole-genome bisulfite sequencing

**Table 3:** Average methylation levels of selected HRE-CpGs under normoxia (N) and hypoxia (H) in parental SW480 cells as determined by whole genome bisulfite sequencing (WGBS)

HRE ID	Distance (kb) from TSS of genes	Average methylation levels in SW480 cells	
		*N*	*H*
chr18:21601384	~ 0.7	1.0	0.7
chr3:49905192	~ 1.3	0.95	0.8
chr18:9656652	~ 52	0.95	0.75
chr10:123098647	~ 37	0.94	0.6

HRE: Hypoxia-response element; Kb: Kilobase; TSS: Transcription start site

Interestingly, DNMT3A was also induced by hypoxia to bind to the proximal region of the *TWIST1* promoter as assessed by ChIP-qPCR (second row of panels from the top, Fig. 3a). We further carried out ChIP-Seq analysis of SW480 cells under normoxia and hypoxia conditions with the use of anti-DNMT3A antibody as described in Materials and Methods. Significantly, consensus DNMT3A-binding peaks were found between genomic locations chr7:19118292 (−656) and chr7:19118579 (−943) in the promoter region of *TWIST1* gene in both replicates under hypoxia condition, whereas none of the replicates showed significant DNMT3A-binding under normoxia condition (Fig. 3b). This result was validated by ChIP-qPCR assay (data not shown). In addition, consistent with the ChIP-PCR data of Fig. 3a, the ChIP-Seq analysis in one of the replicates (Hypoxia-1-IP in Fig. 3b) detected DNMT3A-binding around the HRE CpG (−81) in hypoxia-treated SW480 cells. The above data together indicated that DNMT3A might be recruited to the promoter region to participate in the demethylation process, which would facilitate binding of HIF-1α to the demethylated HRE at -81 position (Fig. S5A), which was accompanied by the increase of HIF-1α protein (Fig. S5B). Indeed, the binding dynamics of DNMT3A and HIF-1α of SW480 cells under hypoxia condition for 6 h, 12 h, 18 h, and 24 h demonstrated that binding of DNMT3A to the *TWIST1* promoter could already be detected at 6 h post-hypoxia treatment, whereas HIF-1α binding was detected ~ 12 h later (compare the top two histograms, Fig. 3c).

As expected, the *TWIST1* proximal promoter region adopted an open chromatin state upon hypoxia treatment. We examined several known histone H3 PTMs, including H3-K9/K18Ac, H3K36me3, H3K9me3, and H3K27me3, by ChIP analysis of SW480 cells. The data revealed that active H3-K9/K18Ac (upper panels of Fig. S5C) and H3K36me3 (third row of panels from top of Fig. 3a) signatures were absent in the *TWIST1* proximal promoter under normoxia, but were apparent upon hypoxia treatment. In contrast, while the repressive H3K9me3 mark was observed in the proximal promoter of *TWIST1* under normoxia and it disappeared upon transfer of the cells to hypoxia (lower panels, Fig. S5C), the H3K27me3 repressive mark was absent in the proximal promoter of *TWIST1* under both normoxia and hypoxia conditions (bottom panels of Fig. 3a) although its cellular level was upregulated by hypoxia (Fig. S5D). Notably, active H3-K9/K18Ac mark disappeared whereas the repressive H3K9me3 mark reappeared in the *TWIST1* proximal promoter region under hypoxia after DNMT3A expression was knocked down by shRNA or knocked out by CRISPR/Cas9 editing (Fig. S5C), suggesting that the hypoxia-induced open chromatin structure of *TWIST1* proximal promoter required the presence of DNMT3A.

On the other hand, the data of Fig. 3a indicated that generation of H3K36me3 mark in the *TWIST1* proximal promoter was hypoxia-dependent as expected [26] but DNMT3A-independent. Given the known physical interactions between DNMT3A and H3K36me3/H3K36me2 [44, 45], we checked the kinetics of hypoxia-induced changes to the H3K36me3 signature in the proximal promoter region of *TWIST1*. Interestingly, parallel to chromatin binding of DNMT3A, this histone H3 PTM was also present in the HRE-containing proximal promoter region of *TWIST1* starting at the 6 h time-point of hypoxia treatment (bottom left panel in Fig. 3c), implying recruitment of DNMT3A by H3K36me3 during hypoxia-induced EMT to demethylate the *TWIST1* promoter. We further observed co-occupancy of DNMT3A and H3K36me3 histone marks in the proximal promoter of *TWIST1* by sequential ChIP assay (Fig. 3d), which provided additional support of the recruitment of DNMT3A to the proximal *TWIST1* promoter by the H3K36me3 mark. Finally, it is interesting to note that hypoxia also induced DNMT3A-binding and appearance of H3K36me3 in the proximal promoter of *SNAIL1* gene (data not shown). Our finding of the enrichment of H3K36me3 mark near the HREs of *TWIST1* and *SNAIL1* promoters is in interesting parallel to the increase of H3K36me3 mark at the HRE region of *PDK1* gene in breast cancer cell line SUM159 upon hypoxia treatment [46].

### Participation of DNMT3A in the induction of hypoxia-mediated EMT is not limited to colon cancer cells

**Fig. 4:**
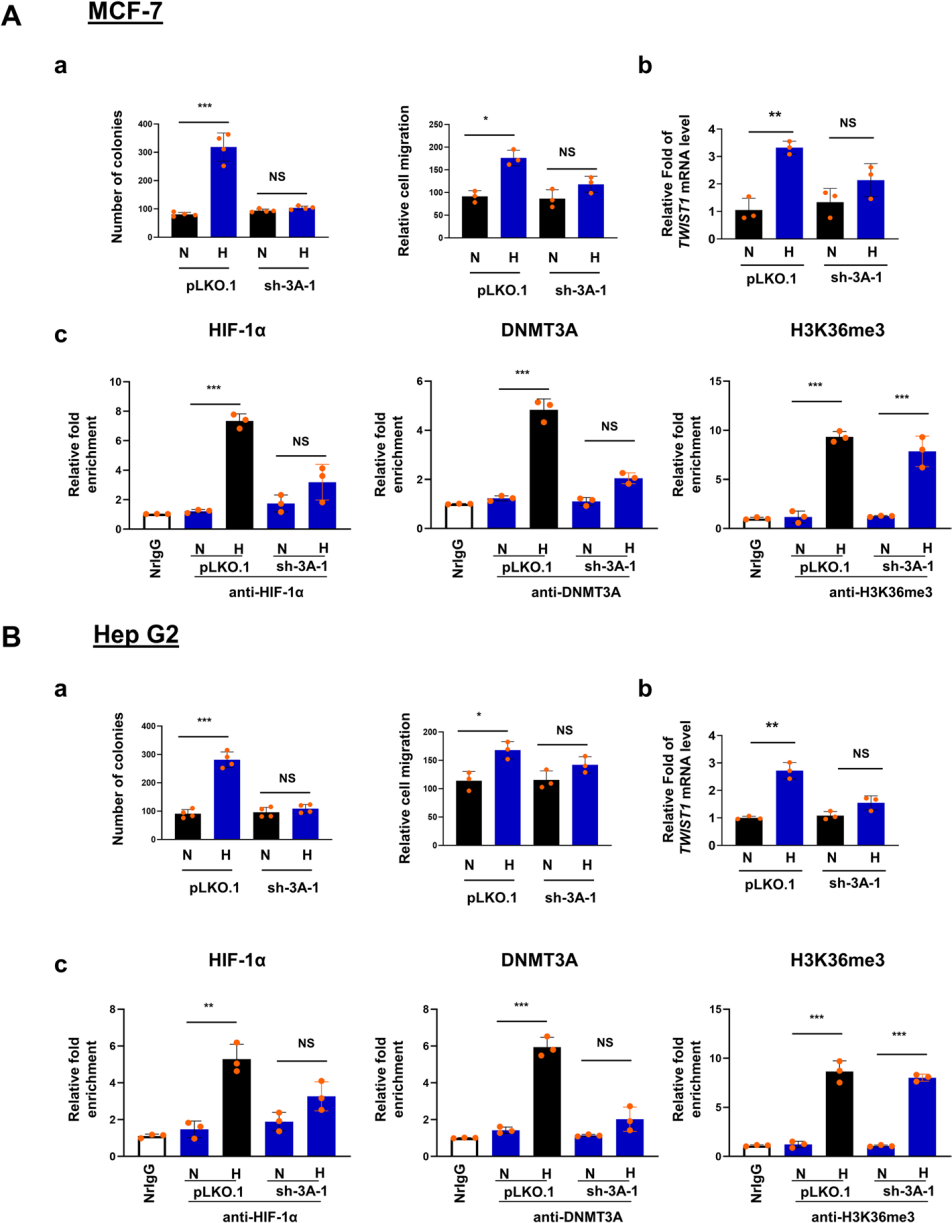
Effects of DNMT3A depletion on hypoxia-induced EMT-associated cellular phenotypes, *TWIST1* transcriptional activation, and enrichment of HIF-1α, DNMT3A and H3K36me3 histone mark in the proximal promoter of *TWIST1* in MCF-7 (**a**) and Hep G2 (**b**) cells. **a** Histograms of the colony formation assay (left) and relative cell migration as determined by the wound-healing assay (right). **b** Hypoxia-induced transcriptional activation of *TWIST1* gene. **c** Patterns of HIF-1α-and DNMT3A-binding as well as enrichment of H3K36me3 histone mark in the proximal promoter region of *TWIST1* gene in cells without (pLKO.1) or with (sh-3A-1) depletion of DNMT3A expression under normoxia (N) or hypoxia (H) conditions. Each histobar represents mean ± SD of 3–4 biological repeats each having three technical repeats. *, **, and *** represent *p* < 0.05, *p* < 0.01 and *p* < 0.005, respectively, by Students *t* test. NS, not significant

We further explored the involvement of DNMT3A in hypoxia-induced EMT process of two other types of cancer cells, namely the breast cancer cell line MCF-7 and hepatocellular carcinoma cell line Hep G2. In both cell lines, shRNA-mediated knockdown resulted in more than 80% reduction of DNMT3A expression (left panels, Fig. S1E). Similar to the SW480 cells, hypoxia treatment caused a robust increase of HIF-1α protein level (right panels, Fig. S1E). Significantly, depletion of DNMT3A by shRNA knockdown also caused impairment of the hypoxia-mediated induction of EMT phenotypes, transcriptional activation of *TWIST1* genes, as well as DNMT3A- and HIF-1α-binding to and enrichment of H3K36me3 histone mark in the proximal promoter region of *TWIST1* gene (Fig. 4). The hypoxia-induced genome-wide DNA demethylation in these two types of cancer cells also appeared to require DNMT3A (Fig. S4). This result indicates that DNMT3A is required for hypoxia-mediated EMT process in multiple types of cancer cells through the binding of HIF-1α to the proximal promoter region and consequent activation of the EMT-associated *TWIST1* gene.

### Requirement of the DNA demethylation activity of DNMT3A for hypoxia-induced EMT of SW480 cells

**Fig. 5:**
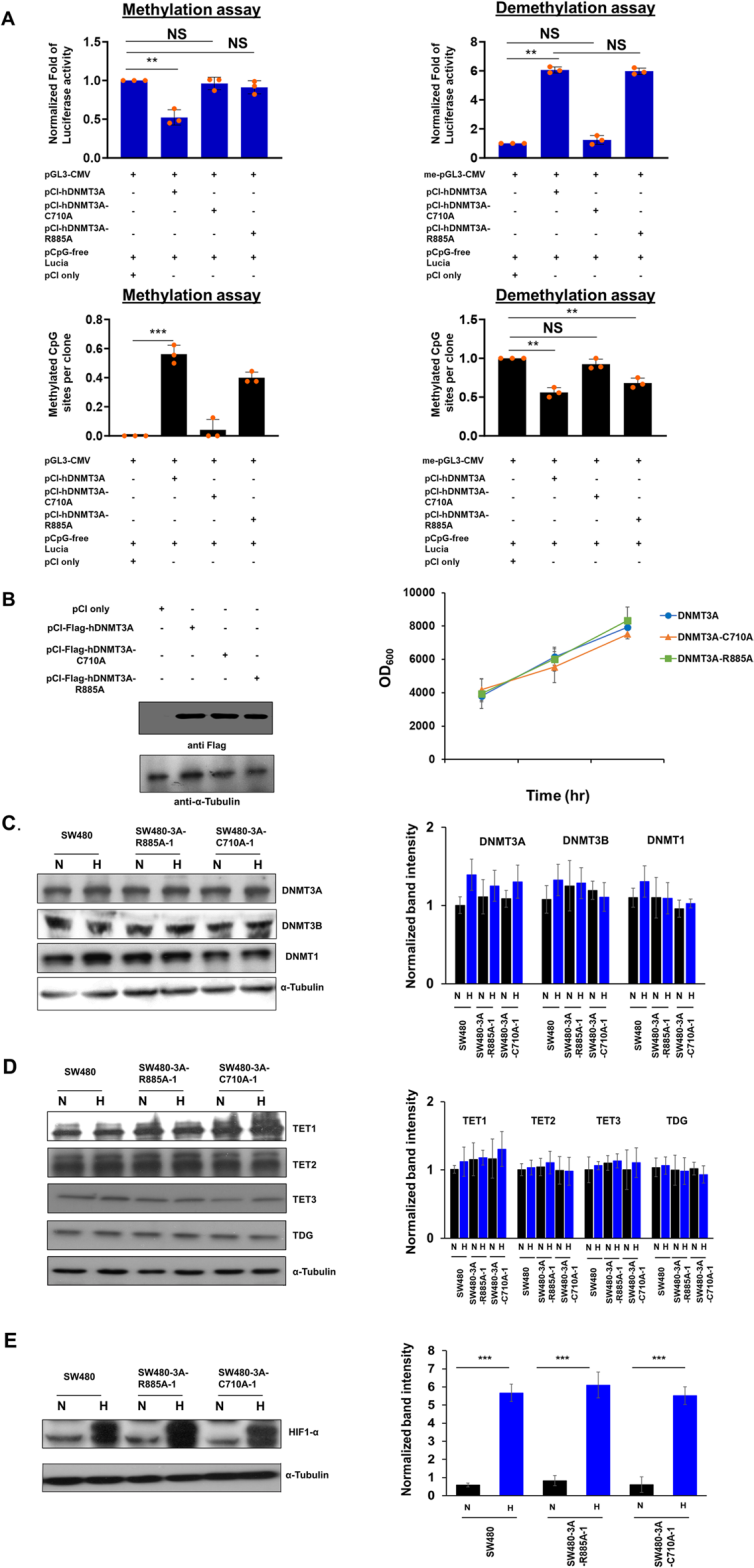
Enzymatic assay of human DNMT3A mutants in a cell-based reporter system and western blotting analysis of CRISPR-Cas9-edited knock-in mutant SW480 cells. **a** and **b** Comparison of the DNA methylation and demethylation activities of mutant and wild type human DNMT3A enzymes in transfected HEK 293 cells. **a** Top, HEK 293 cells were co-transfected with empty pCI vector, pGL3-CMV (left panel, methylation assay), or methylated pGL3 (right panel, demethylation assay), together with pCI-hDNMT3A, pCI-hDNMT3A-C710A, or pCI-hDNMT3A-R885A expression plasmids for 48 h, followed by measurement of Lucia and firefly luciferase activities. Each histobar represents the mean ± SD fold-change of [Firefly/Lucia] activity relative to the control lacking co-expression of hDNMT enzymes. Three independent biological experiments each having three technical repeats were conducted. ** and *** represent *p* < 0.01 and *p* < 0.005, respectively, based on Students *t* test; NS, not significant. Bottom, Na-bisulfite DNA sequencing analysis of the region spanning positions −105 to −307 of pGL3-CMV reporter plasmid DNA recovered from the transfected HEK 293 cells as described above. The mean numbers (± SD) of methylated or demethylated CpG sites per clone from three independent biological experiments are shown. ** and *** represent *p* < 0.01 and *p* < 0.005, respectively, based on Students *t* test; NS, not significant. **b** Similar proliferation rates of HEK 293 cells ectopically overexpressing human wild type DNMT3A, DNMT3A-C710A, and DNMT3A-R885A, respectively. Left, western blots comparing the expression levels of Flag-tagged human wild type DNMT3A, DNMT3A-C710A, and DNMT3A-R885A in transfected HEK 293 cells. The right panel shows the effects of ectopic overexpression of these enzymes on the proliferation of HEK 293 cells at 12 h, 24 h, and 48 h post-transfections. The absorbance values at 600 nm from six independent wells at each time point were averaged and compared. **c**–**e** Comparison of the endogenous expression levels of DNMT3A, DNMT3B, DNMT1, TET protein isoforms, TDG, as well as hypoxia-induced HIF-1α protein in CRISPR/Cas9-edited SW480 knock-in cells. Left panels, western blots showing the expression patterns of the endogenous DNMT isoforms (**c**), TET isoforms and TDG (**d**), and HIF-1α (**e**) in wild type SW480, SW480-3A-R885A-1 and SW480-C710A-1 cells under normoxia (N) and hypoxia (H) conditions. Histograms comparing the levels of the respective proteins are displayed on the right

In view of the above-described results, we investigated that whether the active 5-mC demethylase activity of human DNMT3A was involved in the regulation of *TWIST1* promoter demethylation and transcription during hypoxia-induced EMT of SW480 cells. To accomplish that, we generated several human DNMT3A mutants each of which carrying a single-amino acid substitution, and tested their DNA methylation as well as demethylation capabilities by reporter assay in transfected HEK 293 cells (Fig. 5). Among them and as expected from previous studies of mouse DNMT3A [13], the C710A mutation at the catalytic site for DNA methylation of human DNMT3A abolished its DNA methylation as well as demethylation activities (exemplified in Fig. 5a). Importantly, the R885A mutation of human DNMT3A—the orthologous amino acid (R881) of which in mouse DNMT3A preceding motif X was reported to be required for SAM-binding and catalysis of DNA methylation [47]—abolished its DNA methylation, but not DNA demethylation, capability (Fig. 5a). Both of these mutants were expressed at similar levels and they did not affect the proliferation rate of HEK 293 cells (Fig. 5b).

**Fig. 6:**
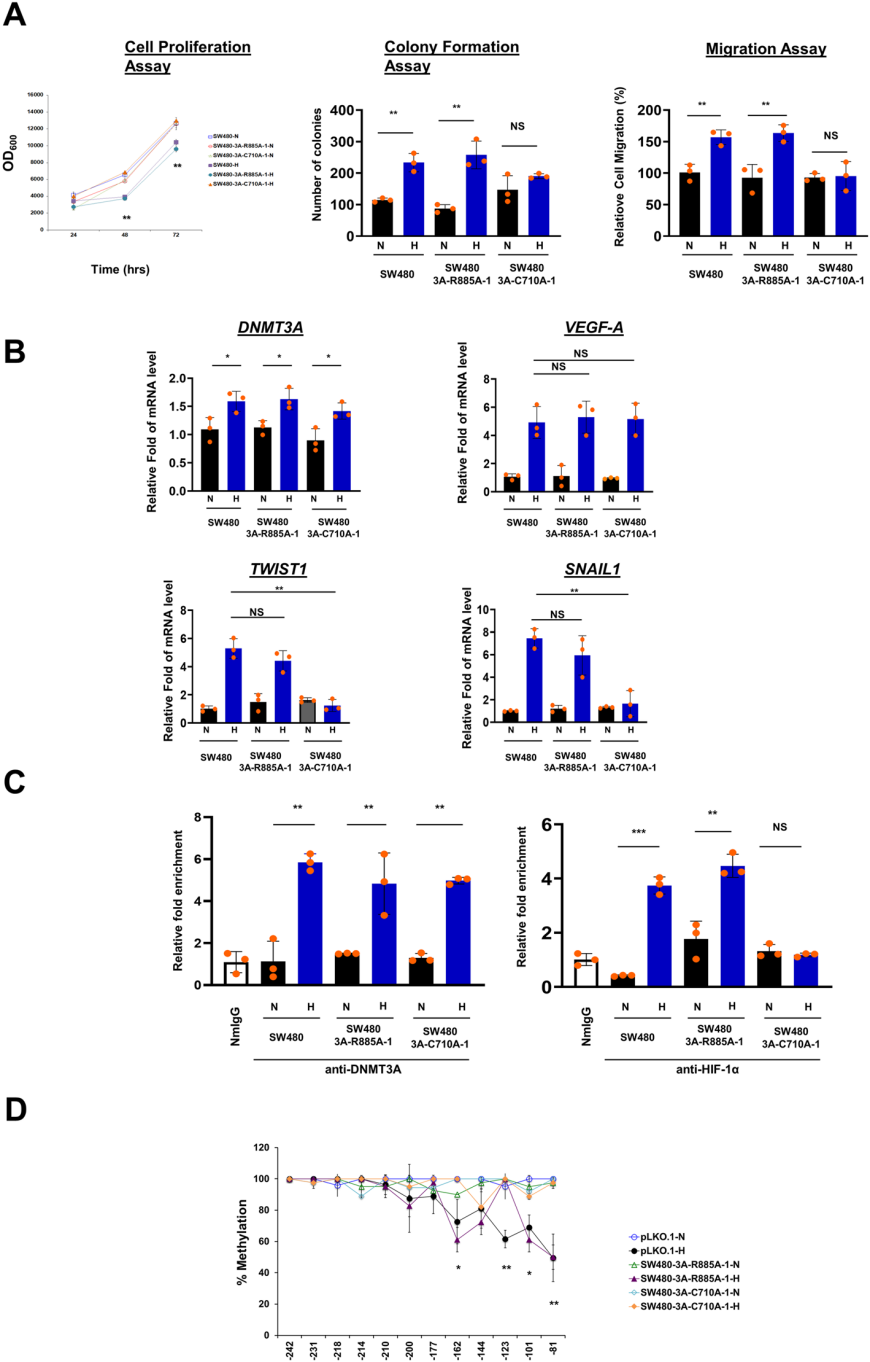
Role of DNA demethylation activity of DNMT3A during hypoxia-induced EMT of SW480 cells. **a** Growth kinetics, colony formation ability and migration capacity of SW480 cells with or without homozygous DNM3A-C710A or DNMT3A-R885A mutation and grown under normoxia (N) or hypoxia (N). All measurements are plotted as mean ± SD from three or more independent biological replicates each having three or more technical repeats. **b** mRNA levels for the *DNMT3A*, *VEGF-A*, *TWIST1* and *SNAIL1* genes in wild type and mutant DNMT3A-harboring SW480 cells grown under normoxia (N) or hypoxia (H). The histobars represent mean ± SD from three to four independent biological replicates each having three or more technical repeats. **c** Binding of HIF-1α and DNMT3A to the *TWIST1* proximal promoter region in parental and mutant DNMT3A-harboring SW480 cells grown under normoxia or hypoxia, as revealed by ChIP assay. ChIP DNA enrichments are expressed as fold-change relative to enrichment in NrIgG pulldown samples, and are represented as mean ± SD from three independent biological replicates each having three or more technical repeats. **d** Methylation profiles for the *TWIST1* promoter in wild type and mutant DNMT3A-harboring SW480 cells grown under normoxia or hypoxia condition, as revealed by Na-bisulfite sequencing analysis. For (**a**–**d**) *, **, and *** represent *p* < 0.05, *p* < 0.01 and *p* < 0.005, respectively, based on Students *t* test; NS: not significant

Next, we used CRISPR/Cas9 editing to generate SW480 cell lines carrying homozygous C710A or R885A mutation and compared their molecular/cellular properties to wild type SW480 cells under normoxic versus hypoxic conditions. Both of the CRISPR/Cas9-edited cell lines presented similar levels of DNMT enzymes (Fig. 5c), and TET isoforms as well as TDG (Fig. 5d), and they were found to have HIF-1α induced by hypoxia (Fig. 5e). As shown in Fig. 6a, SW480-3A-R885A cells, but not SW480-3A-C710A cells, exhibited EMT phenotypes upon hypoxia treatment, including decreased cell growth, increased colony formation, and increased migration-capability. Similar to wild type SW480 cells, the hypoxia-induced EMT of SW480-3A-R885A cells were accompanied by upregulation of *VEGF-A*, *TWIST1*, *SNAIL1*, as well as *DNMT3A* gene expression (Fig. 6b). Remarkably, ChIP-qPCR revealed that similar to wild type DNMT3A in SW480 cells, hypoxia also induced binding of the two mutant forms of DNMT3A to the HRE-containing proximal promoter of *TWIST1* in both SW480-3A-C710A and SW480-3A-R885A cells (left histobar diagram, Fig. 6c). However, only in SW480-3A-R885A cells, but not in SW480-3A-C710A cells, was the *TWIST1* promoter bound with HIF-1α (right histobar diagram of Fig. 6c) and also demethylated upon hypoxia treatment (Fig. 6d). Again, the EMT-associated phenotypes and upregulation of *VEGF-A*, *TWIST1*, *SNAIL1*, and *DNMT3A* genes were similar between the parental SW480 cells and SW480 cells without successful C710A knock-in (data not shown), indicating the absence of CRISPR/Cas9-associated off-target effects.

## Discussion

The EMT program of cancer cells is induced/regulated by a range of conditions including hypoxia [16, 48] and it is associated with changes of epigenetic modifications of the chromatin including DNA methylation and histone post-translational modifications [49]. The cancer cells are responsive to these heterotypic EMT-inducing signals owing to the various genetic and epigenetic alterations they harbor during the primary tumor formation [48]. Hypoxia-mediated destabilization of cellular ROS balance can lead to induction of EMT process in cancer cells [50, 51], which carry high concentration of Ca^2+^ [52]. In interesting connection, previous studies have shown the requirement of Ca^2+^ for the active 5-mC-demethylation activity of mammalian DNMTs, which is enhanced by the H_2_O_2_-induced oxidized environment [13]. Moreover, hypoxia-induced ROS imbalance would generate epigenetic alterations such as the increase of H3K36me3 histone mark owing to the reduction of activity of jmjC domain containing KDM4 family of histone demethylases [28]. Notably, hypoxia-induced reduction in enzymatic activity of jmjC domain containing histone demethylase can also happen in a ROS-independent manner owing to scarcity of oxygen, which acts as a cofactor for their enzymatic activity [26]. In this study, we have explored and established, for the first time, the functional role of the DNA demethylation activity of DNMT3A in the hypoxia-induced EMT of cancer cells through shaping of the DNA methylation landscape of the cancer cell genome and consequent transcriptional activation of essential EMT genes. Significantly, this study also connects the O_2_-dependent lysine-specific histone demethylation with DNMT3A-mediated active 5-mC demethylation during the hypoxia-induced EMT process.

Regulation of the hypoxia-induced EMT of cancer cells is known to involve the TET dioxygenases. In the lung cancer cell line H1299, TET1 is required for the expression of mesenchymal genes *N*-cadherin and vimentin as well as for the expression of INSIG1, a master regulator of cholesterol biosynthesis that drives hypoxia-induced EMT in a catalytic-site independent manner [53]. Furthermore, in breast cancer cell lines MCF-7 and MDA-MB-231, shRNA-mediated depletion of TET1 or TET3 would reduce the expression of mesenchymal genes *N*-cadherin and vimentin during hypoxia. In addition, although hypoxia reduces TET enzymatic activity in cancer cells [54, 55], hypoxia-induced increase of the 5-hmC level locally in the genomic region containing *TNF-α* gene of breast cancer cells is mediated by TET1 and TET3 [56]. On the other hand, the tumor suppressive and oncogenic functions of DNMTs have been reported in relation to the EMT process. For example, in prostate cancer cells DNMT1 was shown to play a tumor-suppressive role [57] while DNMT3B displayed oncogenic function by inducing EMT-associated phenotypes in head and neck squamous cell carcinoma, breast cancer, and ovarian cancer cells [58–60]. Also, DNMT3A exerts oncogenic functions in several cancers. In particular, the DNMT3Ab isoform has been shown to repress expression of *E-cadherin* gene by hypermethylation of its promoter resulting in the induction of TGF-β-mediated EMT in gastric cancer cells [61]. On the other hand, DNMT3Aa induces malignant transformation of lung adenocarcinoma cells by induction of the level of HDAC7 with concomitant increase of ZEB1 and *c*-Myc [62]. However, whether and how DNMTs are involved in the regulation of hypoxia-induced EMT and changes of the methylome of cancer cells have remained unknown until now.

As shown above, we have found that DNMT3A is required for hypoxia-induced EMT (Fig. 1) and activation of *TWIST1* gene of SW480 cells (Figs. 2 and 6). More importantly, the enzymatic activity of DNMT3A functions during hypoxia to demethylate the genomic DNA (Fig. S4) including the *TWIST1* (Figs. 2c and 6d) and *SNAIL1* promoter (data not shown).The upregulation of *TWIST1* gene by DNMT3A-mediated promoter DNA demethylation in SW480 cells (Figs. 2c and 6d) is in correlation with the inverse relationship between the expression level and promoter methylation state of *TWIST1* gene in colon cancer cell lines [31]. Notably, inhibition of DNA replication and consequently passive DNA demethylation by aphidicolin [63, 64] does not block the hypoxia-induced transcriptional activation of *TWIST1* (data not shown). In interesting contrast, we did not find significant change of the expression levels of TET proteins in the colon cancer cell SW480 upon hypoxia (upper panels, Fig. S1C). Furthermore, knockdown of the expression of *TET2* or *TET3* mRNAs in SW480 cells does not affect the hypoxia-induced *TWIST1* activation. On the other hand, knockdown of *TET1* mRNA does reduce the hypoxia-induced increase of *TWIST1* mRNA to some extent (Fig. S3A), suggesting that TET1 is a coactivator of hypoxia-induced transcriptional activation of *TWIST1* gene. However, the hypoxia-induced DNA demethylation of the *TWIST1* promoter or the SW480 genome appears to be independent of TET1 and TET2 (Figs. S3B and S3C), which is in line with the fact that tumor hypoxia would reduce the TET enzymatic activity [54, 55]. Thus, DNMT3A appears to be a major player for the hypoxia-induced EMT process of the colon cancer cells via DNA demethylation, by its DNA demethylation activity, of the SW480 genome including the promoters of essential EMT associated genes like *TWIST1* and *SNAIL1*. It should be mentioned here that the hypoxia condition is known before to induce genomic DNA demethylation globally in human colon cancer cell lines HCT116, DLD-1 and SW480, melanoma cell line WM115, and dermal fibroblast [65]. In addition, the mouse xenografts of human SW480 and WM115 cells exhibits global genomic DNA demethylation when compared with implanted cells [65]. Finally, in interesting parallel, knockdown of DNMT3A also would prevent hypoxia-induced EMT, transcriptional activation of *TWIST1* gene, and global genomic 5mC demethylation in the breast cancer cells (MCF-7) and the hepatocellular carcinoma cells (Hep G2) (Figs. 4 and S4).

**Fig. 7:**
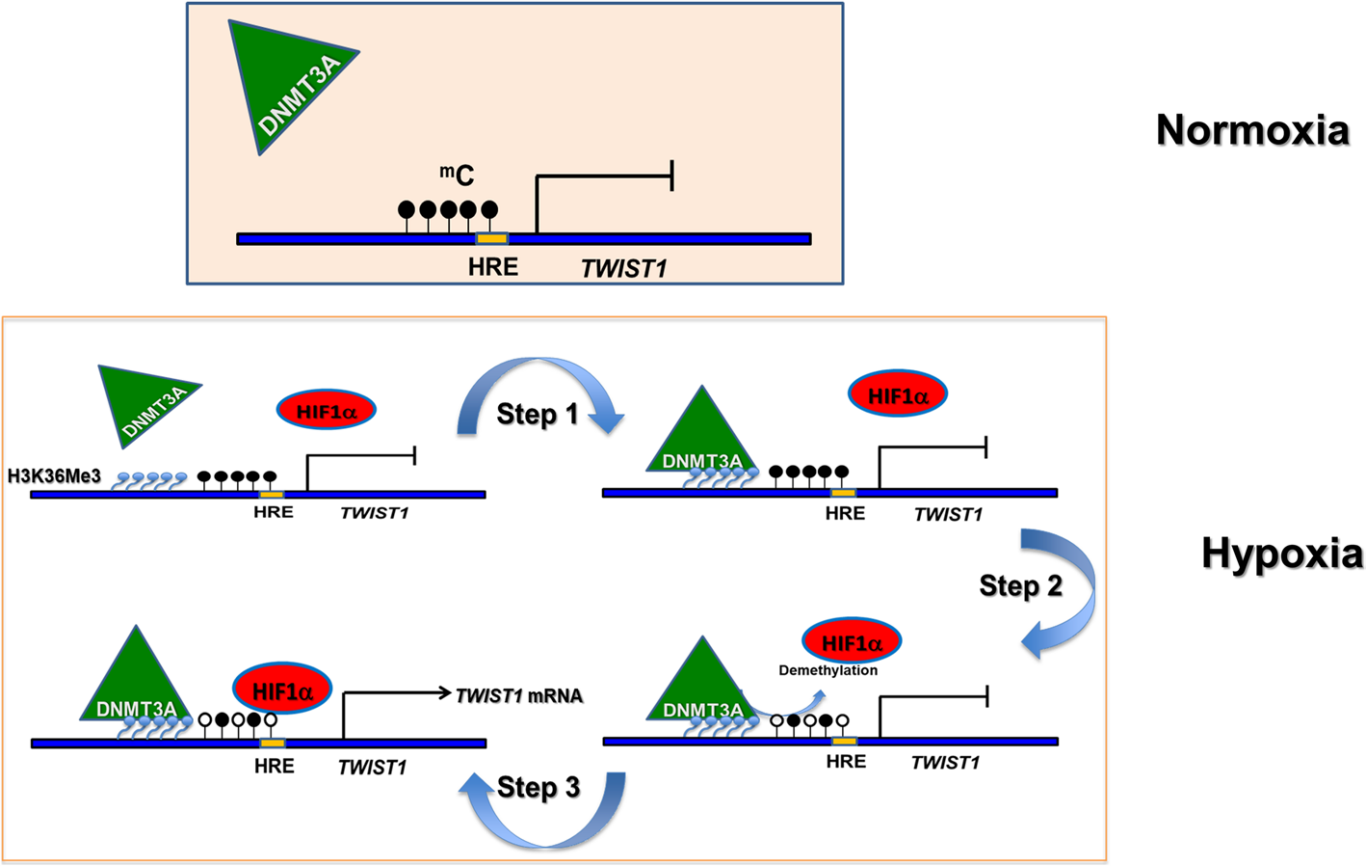
A model depicting the epigenetic regulation of hypoxia-induced EMT of cancer cells by the DNA demethylation activity of DNMT3A. As illustrated, the H3K36me3 histone mark is enriched in the proximal promoter region of the *TWIST1* gene during early phase of hypoxia (step 1), followed by DNMT3A recruitment (step 2). Recruited DNMT3A then demethylates 5-mC (filled black circles) under hypoxia (step 3), thereby facilitating binding of HIF-1α to HRE in the proximal promoter to initiate transcription of the *TWIST1* gene. For more details, see text

Mechanistically, as illustrated in Fig. 7, it appears that one of the early events of hypoxia-induced EMT in cancer cells is the generation of specific histone PTM signatures in the regulatory elements of a subset of EMT-related genes. As exemplified by our analysis of the proximal promoter of the *TWIST1* gene, H3K36me3 represents one such hypoxia-induced histone signature, which is manifested upon reduced activity of one or more of its demethylases arising from low O_2_ conditions [26]. H3K36me3 then recruits DNMT3A to the nearby genomic regions, including the *TWIST1* promoters via their known physical interaction [44, 45]. Following recruitment, the chromatin-bound DNMT3A preferably demethylates CpGs in its vicinity including the HRE CpG under the relatively high Ca^2+^ ion concentration of cancer cells [66]. Once these CpGs in the proximal promoter regions harboring a HRE have been demethylated, HIF-1α binds to the HRE and activates gene transcription. Notably, the occurrence of H3K36me3 mark outside the gene body, as observed by us in this study and by Zhang et al. [67] before, is in contrast to findings in many other genes [68], but the mechanisms remain unclear. The mechanistic scenario presented in Fig. 7 is supported by our results showing the kinetics of DNMT3A and HIF-1α binding to the proximal *TWIST1* promoter and enrichment of H3K36me3 mark in this region of SW480 cells (Fig. 3c) as well as MCF-7 and Hep G2 cells (Fig. 4). Our sequential ChIP assay also points to the H3K36me3-mediated recruitment of DNMT3A to the proximal *TWIST1* promoter (Fig. 3d) in SW480 cells. Interestingly, requirement of DNMT3A for the hypoxia-induced activation of genes, such as *TWIST1* and *SNAIL1* (Fig. 2a), appears to be regulated by the mechanistic scenario presented in Fig. 7, but this scenario does not seem to operate in case of *GLUT1* that fails to show DNMT3A dependence during its hypoxia-mediated activation (Fig. 2a). We also note here that demethylation of *TWIST1* promoter under hypoxia is unlikely to be due to occlusion of DNMT1 in the maintenance DNA methylation process by H1F-1α-binding at HRE (−81 position), since this HRE is heavily methylated under normal condition (Figs. 2c, 6d and Table 2) and it is known in literature [29] and shown by our data (Fig. S5A) as well that methylated HRE repels binding by H1F-1α factor.

Unlike the SW480-3A-C710A cells, the removal of the DNA methylation activity, but not the DNA demethylation activity, of DNMT3A in SW480-3A-R885A cells could still allow the cells to undergo hypoxia-induced EMT program (Fig. 6). Thus, it is unlikely that methylation activity of DNMT3A directly interacts or cooperates with its demethylation activity while activating the EMT program under hypoxia, but participation of other cofactors or epigenetic modifiers in this process cannot be excluded. Moreover, the role of DNMTs as 5mC DNA demethylase depends on the redox state and [Ca^2+^] [13], which are known to influence the chromatin architecture globally or locally [69, 70]. Since establishment of either of these two conditions across the genome likely takes place through multiple mechanistic pathways; it is possible that different cofactors/enzymatic pathways would operate to establish different redox/[Ca^2+^] conditions across the genome and ensure the dominance of the demethylation activity of DNMT3A over its methylation activity at specific genomic loci.

Several other points are worthy to note here. First, the binding of HIF-1α to specific HREs requires both a threshold extent of demethylation of CpGs in the HRE(s) and a threshold level of HIF-1α factor, as exemplified for the *TWIST1* promoter under the hypoxia condition. However, this scenario would also depend on other factors/cellular pathways such as those affecting the histone modifications and chromatin structure [71]. Secondly, although our data convincingly indicate that DNA 5mC demethylation activity of DNMT3A regulates the hypoxia-induced EMT program in cancer cells by direct demethylation of EMT gene promoters including *TWIST1* and *SNAIL1*, the involvement of other signaling pathway(s) mediated by this DNA demethylation activity could not be ruled out at the present time. Finally, knockout of the expression of the three isoforms of DNMTs, individually or simultaneously, by genomic perturbation is known to lead to global reduction of the DNA methylation levels whereas certain genomic regions exhibited higher DNA methylation level in several different cellular systems [72–75]. Noteworthy here is that although DNMT3B is known to partially compensate for the decrease of DNA methylation upon DNMT3A loss during hematopoietic stem cell differentiation [76], it appears that neither DNMT3B nor other factors would compensate for the loss of the wild type DNMT3A with respect to the hypoxia-induced EMT and *TWIST1* promoter activation in SW480 cells (Figs. 1, 2 and 6) as well as MCF-7 and Hep G2 cells (Fig. 4), the demethylation of *TWIST1* promoter in SW480 cells (Figs. 2 and 6), the global genomic 5mC demethylation (Fig. S4) or HIF-1α binding to the *TWIST1* promoter in SW480 cells (Figs. 3 and 6) as well as MCF-7 and Hep G2 cells (Fig. 4). Whether the enzyme also directly demethylates particular loci of genomic DNA under specific conditions, e.g., hypoxia, and whether it could compensate for the increase of DNA methylation at specific genomic loci due to loss of DNMT3A require future investigation.

## Conclusions

Altogether, this study has provided the first demonstration of a physiological function of the active DNA demethylation activity of the DNMTs, particular that of DNMT3A. Equally important, our findings have revealed a missing link between the HIF-1α pathway and the O_2_-sensing KDM pathway, both of which are known to be essential for a wide set of normal and disease-associated cellular processes. With respect to the latter, while the relative contributions of DNMTs and TET enzymes to the epigenetic regulation of tumorigenesis of different types of cancers and the associated regulatory mechanisms await detailed investigation in future, the active DNA demethylation activity of DNMT3A has now emerged as a new potential target for therapeutic development to prevent EMT and metastasis of cancer cells.

## Supplementary Information

Additional file 1.

Additional file 2.

Additional file 3.

Additional file 4.

Additional file 5.

Additional file 6.

Additional file 7.

## Data Availability

The authors declare that all relevant data are included either in the main text or in the supplementary information. The whole-genome bisulfite sequencing and ChIP-Seq data have been uploaded to the Gene Expression Omnibus Series under the accession number GSE252167.

## Abbreviations

EMT: Epithelial-to-mesenchymal transitions
DNMTs: DNA methyltransferases
5-mC: 5-Methylcytosine
PTMs: Post-translational modifications
NER: Nucleotide excision repair
BER: Base excision repair
ROS: Reactive oxygen species
jmjC: Jumonji C
HIFs: Hypoxia-inducible factors
HRE: Hypoxia-response element
FAM: Fluorescein amidites
NrIgG: Normal rabbit IgG
EMSA: Electrophoretic mobility Shift assay
SD: Standard deviation
WGBS: Whole-genome bisulfite sequencing
