# Supplementary figures and images for: Hypoxia-induced genome-wide DNA demethylation by DNMT3A and EMT of cancer cells
Source: Cell Mol Biol Lett. 2025 Aug 5;30:95. doi: 10.1186/s11658-025-00775-x (PMC12326847; doi:10.1186/s11658-025-00775-x)

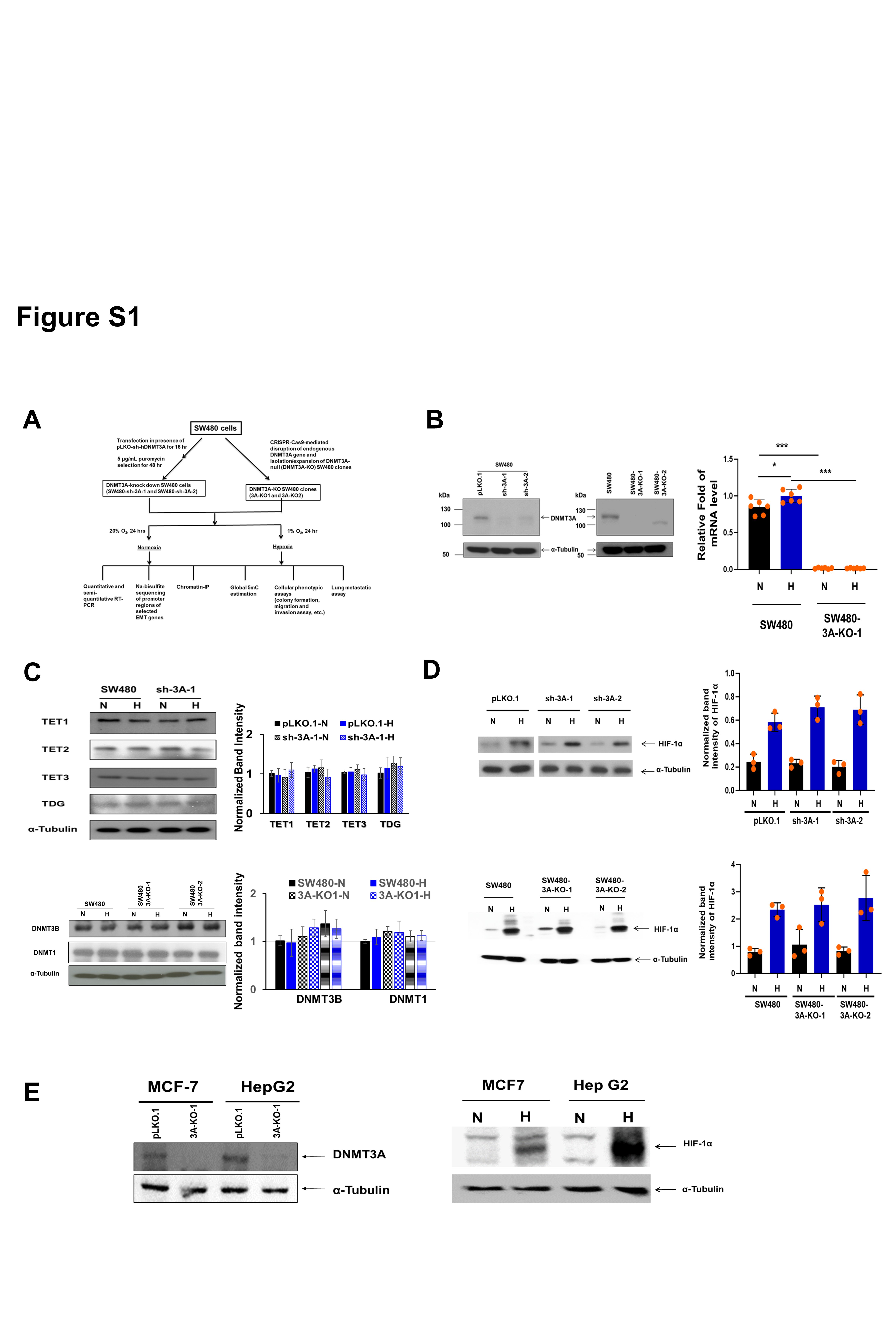

Supplement: Supplementary file 1 — Additional file 1. [file 11658_2025_775_MOESM1_ESM.tif]

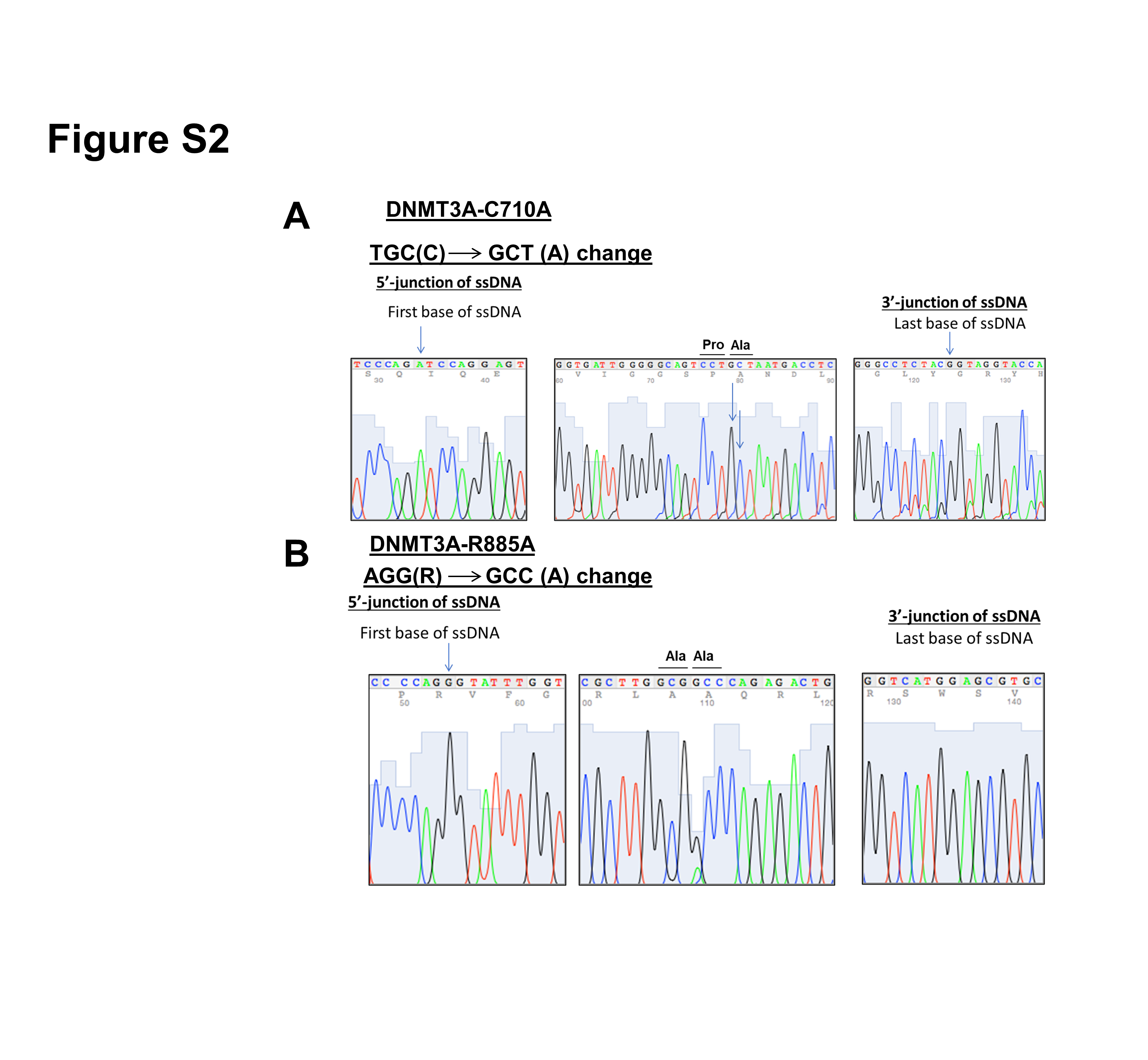

Supplement: Supplementary file 2 — Additional file 2. [file 11658_2025_775_MOESM2_ESM.tif]

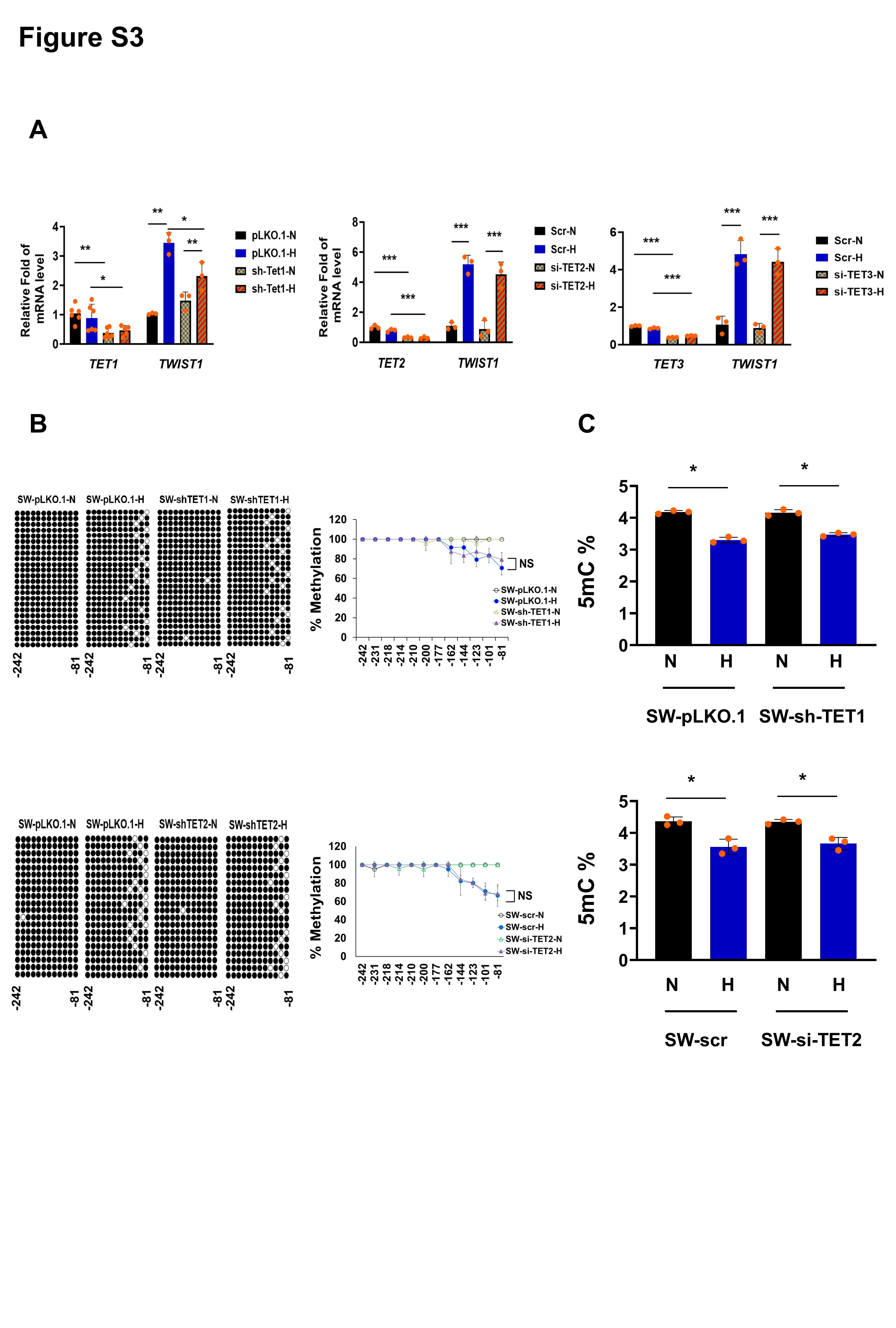

Supplement: Supplementary file 3 — Additional file 3. [file 11658_2025_775_MOESM3_ESM.tif]

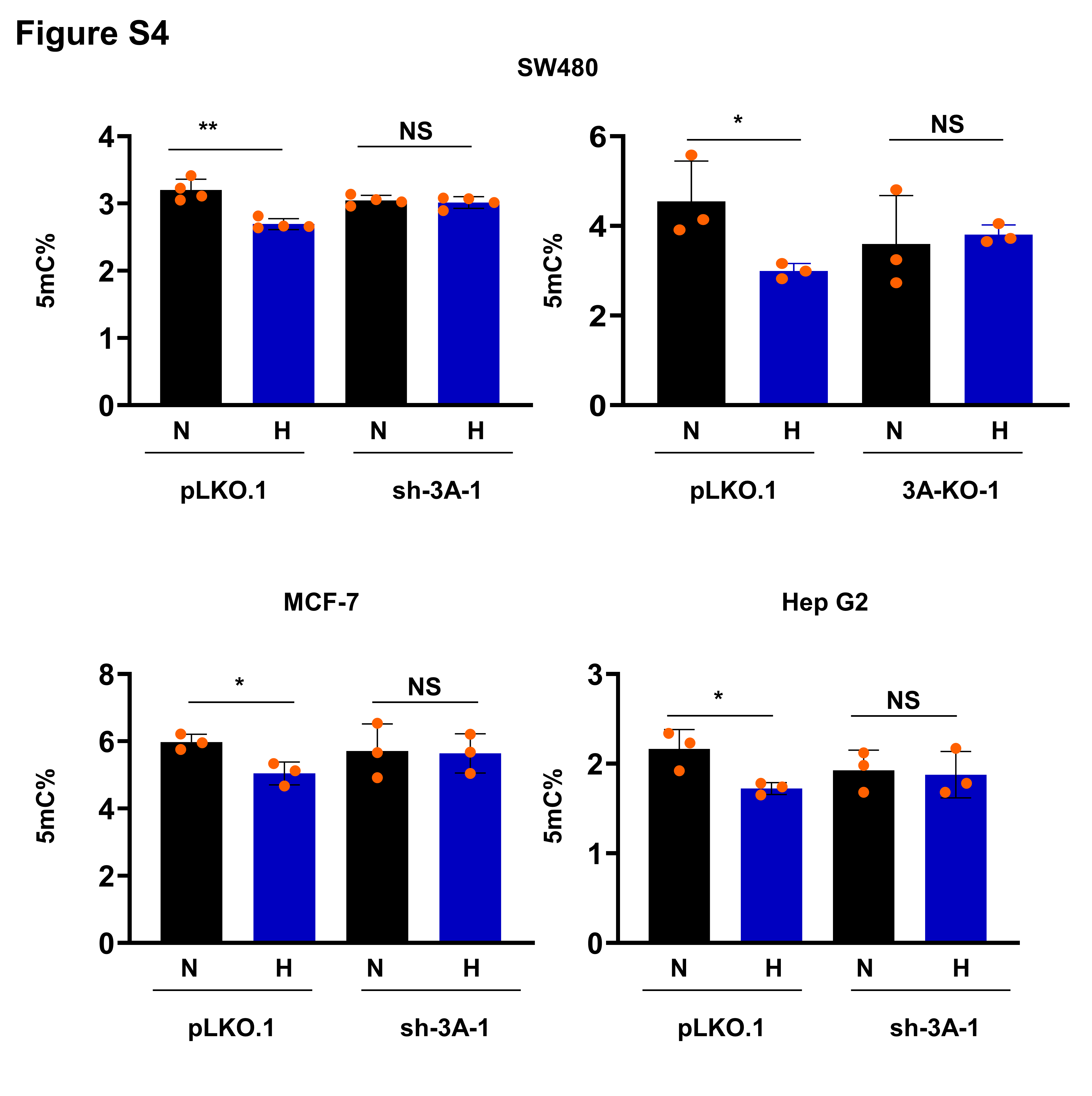

Supplement: Supplementary file 4 — Additional file 4. [file 11658_2025_775_MOESM4_ESM.tif]

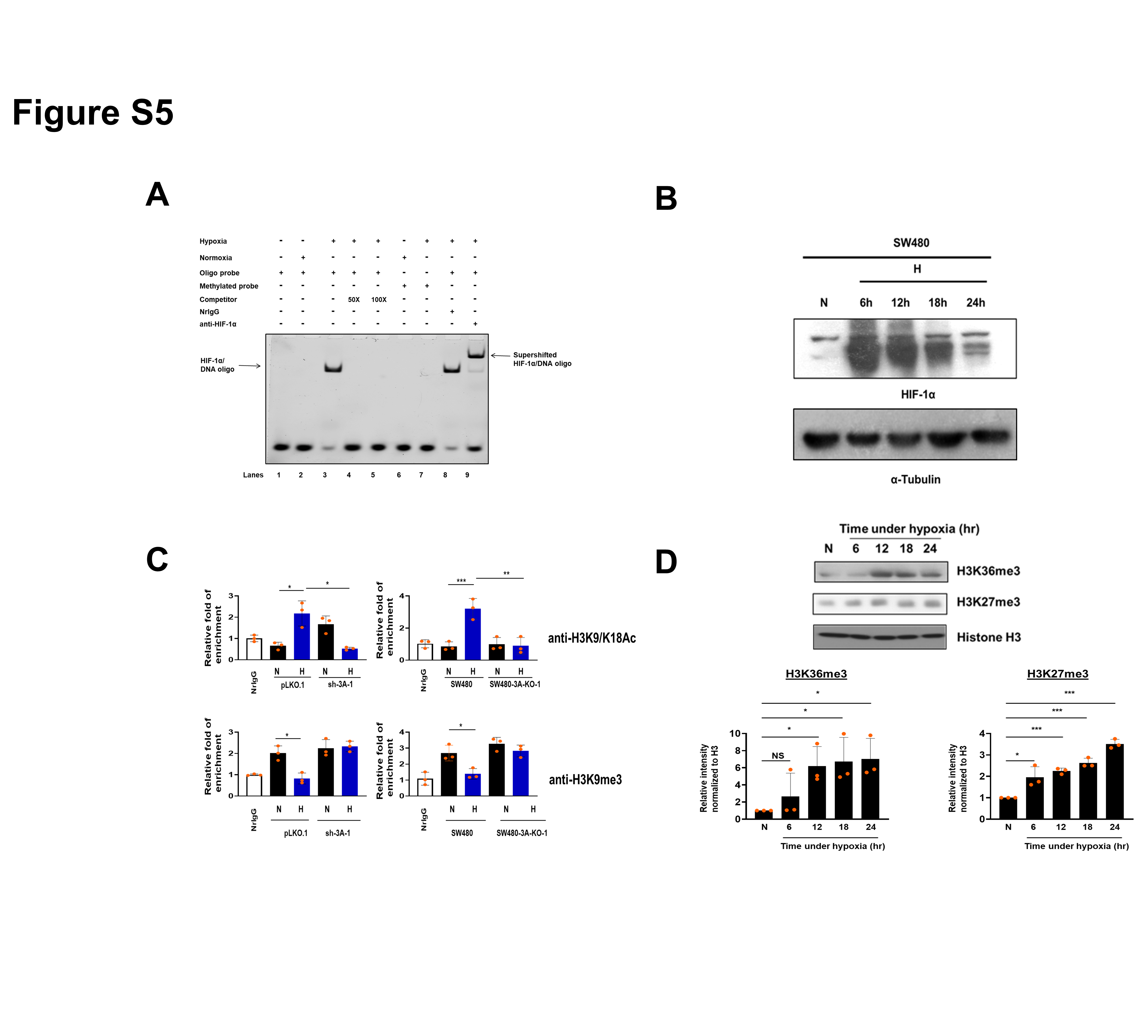

Supplement: Supplementary file 5 — Additional file 5. [file 11658_2025_775_MOESM5_ESM.tif]

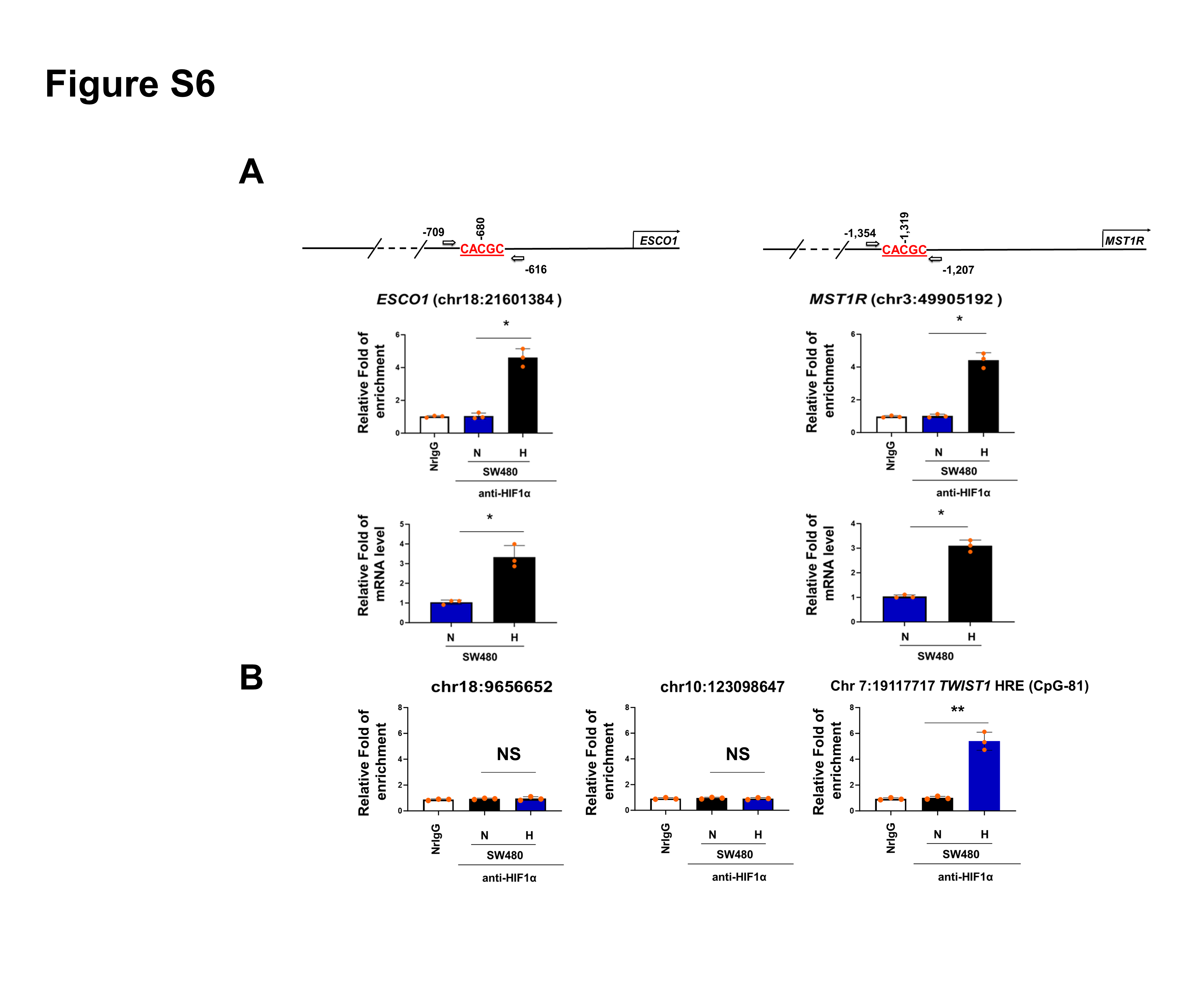

Supplement: Supplementary file 6 — Additional file 6. [file 11658_2025_775_MOESM6_ESM.tif]
