# Supplementary material for: Hypoxia-induced genome-wide DNA demethylation by DNMT3A and EMT of cancer cells
Source: Cell Mol Biol Lett. 2025 Aug 5;30:95. doi: 10.1186/s11658-025-00775-x (PMC12326847; doi:10.1186/s11658-025-00775-x)
Supplement: Supplementary file 7 — Additional file 7. [file 11658_2025_775_MOESM7_ESM.docx]

**Supplementary Information**

**Hypoxia-Induced Genome-wide DNA Demethylation by DNMT3A and EMT of Cancer Cells**

Biswanath Chatterjee^1,^ ^2, §^, Pritha Majumder^1, §^, Chun-Chang Chen^2^, Jing-Ping Wang^2, 3^, Po-Hsuan Su^4^, Hung-Cheng Lai^4^, Ching-Chen Liu^2^, Hsin-Nan Lin^2^, Chen-Hsin Albert Yu^2^, Hanna S. Yuan^2^ and Che-Kun James Shen^1, 2, *^

^1^The PhD Program in Medical Neuroscience, Taipei Medical University, 12F, Education & Research Building, Shuang-Ho Campus, No. 301, Yuantong Road, Zhonghe District, New Taipei city 235, Taiwan.

^2^ Institute of Molecular Biology, Academia Sinica, No. 128, Section 2, Academia Rd, Nangang District, Taipei City, 115, Taiwan.

^3^National Applied Research Laboratories, National Laboratory Animal Center

Building G, No. 111, Lane 130, Section 1, Academia Road, Nangang District, Taipei City 115021, Taiwan

^4^Translational Epigenetics Center, Shuang Ho Hospital, Taipei Medical University, New Taipei City, 235, Taiwan.

^§^ These authors contributed equally.

^*^Corresponding author. Email: [ckshen@gate.sinica.edu.tw](mailto:ckshen@gate.sinica.edu.tw)

**Supplementary figure legends**

**Fig. S1.** (A) Scheme of the experimental strategy to generate SW480 cells with partial or complete depletion of endogenous DNMT3A and subsequent analyses of hypoxia-induced EMT at cellular and molecular levels.

(B) Western blotting analysis of DNMT3A in SW480 cells treated with sh-3A-1 or sh-3A-2 (left panels) and in the two DNMT3A knockout lines SW480-3A-KO-1 and SW480-3A-KO-2 (middle panels). Histogram comparing the *DNMT3A* mRNA levels in parental SW480 and SW480-3A-KO-1 cells under normoxia (N) and hypoxia (H) conditions is shown on the right.

(C) Western blotting analysis of the levels of TET1, TET2, TET3, TDG (top panels) in SW480 and sh-3A-1 treated SW480 cells as well as DNMT3B and DNMT1 (bottom panels) in parental SW480, SW480-3A-KO-1, SW480-3A-KO-2 cells under normoxia (N) and hypoxia (H) conditions. The Western blots are shown on the left and the histograms are shown on the right.

(D) Left panels, Western blots showing the hypoxia-induced increase of HIF-1α in SW480 cells with knockdown of DNMT3A expression by shRNAs and in the two DNMT3A-ablated SW480 clones, respectively. The statistical analysis is shown in the two histograms on the right. The band intensities of HIF-1α were normalized with respect to the cellular α-tubulin under normoxia (N) and hypoxia (H) conditions. For each of the histobars, mean normalized band intensity of HIF-1α ± SD was derived from three or more independent Western blots using ImageJ software (NIH, USA).

(E) Western blots showing the depletion of DNMT3A by sh-3A-1 (left panels) and hypoxia(H)-induced increase of HIF-1α (right panels) in MCF-7 and Hep G2 cells.

**Fig. S2. Validation of DNMT3A knockin mutant SW480 cell lines by DNA sequencing.**

The genomic regions harboring the C710A and R885A mutations were amplified by PCR of the genomic DNAs harvested from SW480-3A-C710A-1 (A) and SW480-3A-R885A-1 (B) cells, respectively, using specific primer sets. The amplicons were then subjected to DNA sequencing. The 5′- and 3′-junctions of the single-stranded donor templates together with the regions containing the indicated mutations are shown in both cases.

**Fig. S3. Failure of depletion of TET isoforms to prevent hypoxia-induced demethylation of the proximal promoter of *TWIST1* gene and its transcriptional activation as well as the global 5mC demethylation in SW480 cells.**

(A) Effects of shRNA-mediated reduction of *TET1* (left histogram) and siRNA-mediated depletion of *TET2* (middle histogram) or *TET3* (right histogram) upon hypoxia-induced *TWSIT1* mRNA level in SW480 cell line under normoxia (N) and hypoxia (H) conditions were analyzed by RT-qPCR. The histobars represent mean ± SD from three or four independent biological experiments each having three technical repeats. *, ** and *** represents p<0.05, p<0.01 and p<0.005, respectively, based on Students *t* test; NS, not significant.

(B) Na-bisulfite DNA sequencing analysis of the *TWIST1* promoter region (positions -81 to -242) of parental SW480 cells under normoxia (N) or hypoxia (H) conditions, as compared to SW480 cells in which TET1 or TET2 was depleted by shRNA-and siRNA-mediated knockdown. The level of methylation at each of the CpG sites is expressed as mean % methylation ± SD from three independent experiments. NS: not significant based on Students *t* test.

(C) Comparison of the genomic 5mC contents (%) of SW480 cells with or without depletion of TET1 (upper histogram) or TET2 (lower histogram). The cells were grown under normoxia (N) or hypoxia (H) condition. Genomic 5mC contents were assayed by HPLC-MS. Each histobar represents the mean ± SD from three different experiments. * and ** represent p<0.05 and p<0.01, respectively, based on Students *t* test; NS, not significant.

**Fig. S4. Hypoxia-induced global 5-mC demethylation of SW480, MCF-7 and Hep G2 cells depends on the presence of DNMT3A.**

Top, comparison of the genomic 5-mC contents (%) of SW480 cells with or without depletion of DNMT3A by shRNA knockdown (sh-3A-1, left panel) or by CRISPR-Cas9-mediated knockout (3A-KO-1, right panel). Bottom, comparison of the genomic 5mCs content (%) of MCF-7 cells (left panel) and Hep G2 cells (right), respectively, with or without depletion of DNMT3A by shRNA knockdown (sh-3A-1). The cells were grown under normoxia (N) and hypoxia (H) condition. Genomic 5-mC contents were assayed by LC-ESI-MS/MS. Each histobar represents the mean ± SD from three independent biological experiments each having three technical repeats. * and ** represent p<0.05 and p<0.01, respectively, based on Students *t* test; NS, not significant.

**Fig. S5.** (A) EMSA analysis of hypoxia-induced HIF-1α binding to HRE. Lane 1, FAM-labeled and HRE-containing DNA oligo probe without incubation with the nuclear extract. Shift of the probe was observed after incubation with nuclear extract from SW480 cells under hypoxia but not normoxia (compare lane 3 to lane 2). No shift could be observed when excess of the unlabeled oligo probe was present in the binding reactions (lanes 4 and 5), or when CpG methylated probe was used (lane 7). Addition of anti-HIF-1α but not the normal rabbit IgG antibody caused supershift of HIF-1α bound non-methylated probe (compare lane 9 to lane 8).

(B) Western blotting to compare HIF-1α levels in parental SW480 and SW480-3A-KO-1 cells under normoxia condition and upon hypoxia treatment for 6 hr, 12 hr, 18 hr and 24 hr.

(C) Comparative ChIP-qPCR assays of hypoxia-induced changes of the appearance of H3K9/K18Ac and H3K9me3 histone marks in the proximal promoter region of *TWIST1* (-144 to +54) for SW480 cells with or without shRNA-mediated (left 2 histographs) or CRISPR/Cas9-mediated (right 2 histographs) depletion of DNMT3A. Each histobar represents the mean ± SD from three independent biological experiments each having three technical repeats.

(D) Top, representative Western blots showing levels of H3K36me3 and H3K27me3 histone marks in SW480 cells at different time-points of hypoxia treatment. Bottom, histographs comparing the relative intensities of H3K36me3 (left) and H3K27me3 (right) normalized to total H3 under normoxia (N) and hypoxia (H) at different time-points. Each histobar represents the mean ± SD from three independent Western blots. *, ** and *** represent p<0.05, p<0.01 and p<0.005, respectively, based on Students *t* test; NS, not significant.

**Fig. S6.** **Hypoxia-induced binding of HIF-1α at the HREs chr18:21601384 and chr3:49905192 but not chr18:** **9656652 and chr10:123098647.**

(A) Top, schematic maps of the *ESCO1* and *MST1R* promoter regions. Middle two panels, the binding of HIF-1α around the HREs chr18:21601384 and chr3:49905192 in the upstream promoter regions of *ESCO1* and *MST1R* genes, respectively, in SW480 cells grown under normoxia (N) and hypoxia (H) conditions was assayed by ChIP-qPCR. The DNA binding of HIF-1α is expressed as fold-change relative to the NrIgG pull-down samples, and expressed as mean ± SD of three independent biological replicates each having three technical repeats. The * represents p<0.05 by Students *t* test. Bottom two panels, total RNAs harvested from SW480 cells were subjected to quantitative RT-PCR assay using primers specific for *ESCO1* and *MST1R* mRNAs. Histobars represent mean ± SD from three independent biological experiments each having three technical repeats. The * represents p<0.05 by Student’s t-test.

(B) Lack of binding of HIF-1α around the HRE-CpGs chr18:9656652 and chr10:123098647 in parental SW480 cells grown under normoxia (N) and hypoxia (H) conditions as assayed by ChIP-qPCR. Parallel analysis of HIF-1α-binding in the *TWIST1* promoter was conducted as the positive control. The DNA binding of HIF-1α is expressed as fold-change relative to the in NrIgG pull-down samples, and expressed as mean ± SD of three independent biological replicates each having three technical repeats. The ** represents p<0.01 by Students *t* test. NS, not significant.
